# Assessment of somatic single-nucleotide variation in brain tissue of cases with schizophrenia

**DOI:** 10.1038/s41398-018-0342-0

**Published:** 2019-01-17

**Authors:** John F. Fullard, Alexander W. Charney, Georgios Voloudakis, Andrew V. Uzilov, Vahram Haroutunian, Panos Roussos

**Affiliations:** 1 Department of Psychiatry, Icahn School of Medicine at Mount Sinai, One Gustave L. Levy Place, New York, NY 10029 USA; 2 Department of Genetics and Genomic Sciences, Icahn School of Medicine at Mount Sinai, Institute for Genomics and Multiscale Biology, One Gustave L. Levy Place, New York, NY 10029 USA; 3 Sema4, 333 Ludlow Street, Stamford, CT 06902 USA; 4 Mental Illness Research, Education, and Clinical Center (VISN 2 South), James J. Peters VA Medical Center, Bronx, NY 10468 USA

## Abstract

The genetic architecture of schizophrenia (SCZ) includes numerous risk loci across a range of frequencies and sizes, including common and rare single-nucleotide variants and insertions/deletions (indels), as well as rare copy number variants (CNVs). Despite the clear heritability of the disease, monozygotic twins are discordant for SCZ at a significant rate. Somatic variants—genetic changes that arise after fertilization rather than through germline inheritance—are widespread in the human brain and known to contribute to risk for both rare and common neuropsychiatric conditions. The contribution of somatic variants in the brain to risk of SCZ remains to be determined. In this study, we surveyed somatic single-nucleotide variants (sSNVs) in the brains of controls and individuals with SCZ (*n* = 10 and *n* = 9, respectively). From each individual, whole-exome sequencing (WES) was performed on DNA from neuronal and non-neuronal nuclei isolated by fluorescence activated nuclear sorting (FANS) from frozen postmortem prefrontal cortex (PFC) samples, as well as DNA extracted from temporal muscle as a reference. We identified an increased burden of sSNVs in cases compared to controls (SCZ rate = 2.78, control rate = 0.70; *P* = 0.0092, linear mixed effects model), that included a higher rate of non-synonymous and loss-of-function variants (SCZ rate = 1.33, control rate = 0.50; *P* = 0.047, linear mixed effects model). Our findings suggest sSNVs in the brain may constitute an additional component of the complex genetic architecture of SCZ. This perspective argues for the need to further investigate somatic variation in the brain as an explanation of the discordance in monozygotic twins and a potential guide to the identification of novel therapeutic targets.

## Introduction

Neural stem cells and neural progenitor cells produce tens of billions of neurons during the development of a healthy human brain^1^, with some estimates suggesting that a developing brain must produce, on average, ~250,000 new neurons every minute^2^. Although remarkably efficient, DNA replication and DNA repair are not flawless processes; with a genome consisting of some 3 × 10^9^ base pairs, errors in DNA replication and repair have been proposed to correspond to ~1.3 errors per cell division in the human brain^3^. In addition to replication and repair errors, non-inherited genetic variants can arise through a myriad of other molecular mechanisms^4^. As such, cells within an individual are genetically heterogenous and may contain an array of non-germline (or, “somatic”) variants, including single-nucleotide variants (SNVs), indels^5^, as well as structural variants, such as copy number variants (CNVs), DNA breaks, inversions, and translocations^6^. Indeed, a study of single neurons from the human prefrontal cortex identified more than a thousand somatic SNVs (sSNVs) per cell^7^. If such mutations occur in critical genes, they may impact the function of the affected cells. Assuming it does not retard cell growth, the earlier a mutation arises during development, the more cells it will affect, and the more likely it is to lead to defects in tissue function and, ultimately, disease ^8^.

The genetic architecture of schizophrenia (SCZ) is highly complex, with risk conferred through common variants^9^, de novo mutations^10^, rare CNVs^11^, and rare SNVs^12,13^. The concordance rate between monozygotic twins is in the range of 41%–65%^14^, suggesting non-inherited factors also make significant contributions to disease risk. Somatic variation is one such potential factor; however, while extensive somatic variation in the brain is now a well-established phenomenon, its relevance to SCZ risk remains unclear. To that end, there is some recent evidence suggesting that somatic variation in the brain may play a role in SCZ, including deletions^15^, CNVs^16^, and long interspersed element-1 (L1) retrotransposons^17,18^. Studies evaluating the contribution of sSNVs in the brain to the genetic architecture of SCZ are lacking.

Here, we report rates of sSNVs in the brain of SCZ cases and controls identified from high-coverage whole-exome sequencing (WES) of neurons and non-neurons isolated by fluorescence activated nuclear sorting (FANS) from frozen postmortem prefrontal cortex (PFC) specimens. Separation of neuronal and non-neuronal nuclei was facilitated by using an established neuronal specific antibody, anti-NeuN^19^, and a protocol that has been extensively used by our team^20,21^. We identified and validated a number of cell-type-specific somatic variants in PFC in SCZ, and found a burden of sSNV in cases compared to controls that included higher rates of non-synonymous sSNVs. Genes affected by SCZ sSNVs were enriched for gene sets that have been implicated previously in SCZ by de novo SNVs and show prenatal-bias expression in human brain during neurodevelopment^22^. Although larger studies are necessary, our findings provide additional evidence that somatic mutations may contribute to SCZ and suggests new avenues of research toward better understanding and treatment of this common disorder.

## Materials and methods

### Sample information

Specimens were obtained from 20 subjects (nine cases, 11 controls) from the Mount Sinai NIH Brain Bank and Tissue Repository (NBTR) (Supplementary Table 1). All subjects were recently included in a large study of gene expression by the CommonMind Consortium^23–25^. In brief, the NBTR obtains brain specimens from the Pilgrim Psychiatric Center, collaborating nursing homes, Veteran Affairs Medical Centers, and the Suffolk County Medical Examiner’s Office. Disease diagnoses are made based on DSM-IV criteria and are obtained through direct assessment of subjects using structured interviews and/or through psychological autopsy by extensive review of medical records and informant and caregiver interviews. Informed consent is obtained from the next of kin. The brain bank procedures are approved by the Icahn School of Medicine at Mount Sinai Institutional Review Board (IRB) and are exempt from further IRB review due to the collection and distribution of postmortem specimens.

### Tissue processing

At autopsy, from each subject fresh frozen slabs were cut from the temporal muscle and Brodmann areas 9/46 of the left dorsolateral prefrontal cortex. Immediately after dissection, specimens were cooled to −190 °C and dry homogenized to a coarse powder using a liquid-nitrogen-cooled mortar and pestle. The tissue was stored at −80 °C until processed.

### FANS of neuronal and non-neuronal nuclei

Fifty milligrams of frozen brain tissue was homogenized in cold lysis buffer (0.32 M sucrose, 5 mM CaCl_2_, 3 mM magnesium acetate, 0.1 mM, EDTA, 10 mM Tris-HCl, pH 8, 1 mM DTT, 0.1% Triton X-100), and filtered through a 40-µm cell strainer. The flow-through was underlaid with sucrose solution (1.8 M sucrose, 3 mM magnesium acetate, 1 mM DTT, 10 mM Tris-HCl, pH 8) and subjected to ultracentrifugation at 24,000 rpm for 1 h at 4 °C. Pellets were re-suspended in 500 µl DPBS supplemented with BSA (at a final concentration 0.1%) and incubated with anti-NeuN antibody (1:1000, Alexa488 conjugated, Millipore cat. #MAB377X) under rotation for 1 h, at 4 °C, in the dark. Prior to FANS sorting, DAPI (Thermoscientific) was added to a final concentration of 1 µg/ml. DAPI positive NeuN+ (neuronal) and NeuN− (non-neuronal) nuclei were sorted into individual tubes, pre-coated with 5% BSA, using a FACSAria flow cytometer (BD Biosciences).

### DNA isolation and sequencing

DNA was isolated from sorted nuclei using the Qiagen QIAamp DNA mini kit (cat. #51306) according to manufacturer’s instructions (Blood or Body fluid spin protocol). Similarly, DNA from temporal muscle samples was extracted using the Qiagen QIAamp DNA mini kit (DNA purification from tissues protocol). Purified DNA was quantified by Qubit (Life technologies) and submitted for WES sequencing (New York Genome Center). Samples were barcoded and pooled prior to enrichment for exonic DNA with the SureSelect Human All Exon V4 library. WES was performed on the HiSeq 2500 platform (Illumina, San Diego, CA, USA), producing 150 base-pair (bp) paired-end reads to a target depth of 250 reads per base for brain specimens and 50 reads per base for temporal muscle.

### Sequence alignment and germline variant calling

To facilitate alignment and germline SNV calling, we utilized a previously described in-house genome analysis pipeline composed from several widely used open source software projects^26^. In brief, short-reads were aligned to a build of the hg19 human reference genome masked for gender and pseudo-autosomal regions using bwa mem^27^. Indel realignment, de-duplication, and base-quality score recalibration (BQSR) were then implemented in accordance with “GATK Best Practices” guidelines. Germline SNVs were called with the GATK HaplotypeCaller^28,29^, and variant quality score recalibration (VQSR) was used to estimate the probability that a WES-identified germline SNV was a true variant instead of an artifact.

Per-individual quality control metrics were calculated from the output of alignment and germline SNV calling procedures. Low level contamination was assessed using VerifyBamID^30^. Coverage metrics derived from the alignment data were calculated using Picard (http://broadinstitute.github.io/picard) and germline SNV metrics derived from the Haplotypecaller output were calculated using PLINK/SEQ^12^. The latter included the total number of alternate alleles, mean heterozygosity, mean chromosome X heterozygosity, dbSNP percentages, and mean transition/transversion ratio at heterozygous sites. All samples were noted to have dbSNP percentages >95%, and all of the other metrics considered displayed broadly even profiles across samples (Supplementary Table 4) and thus were not used as the basis for further individual-level QC.

Identity concordance was performed between the three exomes labeled as being derived from the same individual. This was accomplished in PLINK^31^ using identity-by-state (IBS) and identity-by-descent (IBD) metrics derived from genotypes in a set of ~5000 SNPs in the WES data shown previously to be ancestry informative^12^. These procedures led to the identification of a genetic mismatch between the temporal muscle and brain specimens of one individual, who was therefore excluded from the study. The input into sSNV calling algorithms therefore consisted of three exomes per individual (neurons from the brain, non-neurons from the brain, and temporal muscle) from 19 individuals (nine cases, 10 controls), for a total of 57 exomes.

### Somatic SNV calling and quality control

We called sSNVs using MuTect^32^ (v1.1.6) and Strelka^33^ (v1.0.14) following a comparison of 6 sSNV calling algorithms (Supplementary Information; Supplementary Figures 1–3). One non-neuronal brain specimen was removed due to an excess of sSNVs (Supplementary Information). Only sSNVs called by both algorithms were retained. This set was filtered using a conservative in-house pipeline that kept only those putative sSNVs that met all of the following criteria: mapping quality >10, base quality >10, read depth >10, 2 alleles observed, not a small insertion or deletion, >10 base pairs from another putative sSNV, <350 base pairs outside a target region in the exon capture kit, and minor allele frequency <0.001 (Supplementary Information). The quality and depth filters were required to be met in all three tissues for the individual with the putative sSNV. These initial quality-control procedures removed over 99% of the initial 18,522 sSNV calls made by MuTect and Strelka. The remaining 151 putative variants were then manually inspected with the Integrative Genomics Viewer (IGV; Supplementary Information)^34^. Most (~79%) were determined to be likely artifact based on the manual inspection procedure, leading to a final set of 32 sSNVs for downstream analyses (Supplementary Figure 4).

### Mutational signature analysis

We performed signature analysis by estimating the frequency of mutations in their context for a trinucleotide substitution matrix using sSNVs from the current study (which we call “Fullard” signatures) and two studies conducted in single neurons by Lodato et al.^35^ (“Lodato” signatures) and Bae et al.^3^ (“Bae” signatures). Mutation signatures were detected using the *signeR* package, which applies a Bayesian nonnegative matrix factorization-based mutational signature framework^36^. We run separate analysis in each study (Fullard, Lodato, and Bae) using the default *signeR* parameters. The number of signatures for each dataset was determined based on the maximization of the median Bayesian Information Criterion as implemented in the *signeR* package. We identified 1, 2, and 3 signatures for Fullard, Bae, and Lodato datasets, respectively. The identified signatures were clustered with the 30 COSMIC signatures (http://cancer.sanger.ac.uk/cosmic/signatures), using unsupervised hierarchical clustering with correlation as the distance metric.

### Gene set enrichment analysis

To define gene sets enriched for sSNVs in cases with SCZ compared to controls, we used Mutation Enrichment Gene set Analysis of Variants (MEGA-V; https://github.com/ciccalab/MEGA)^37^. The SCZ and control gene sets were defined based on genes affected by sSNVs. We ran enrichment analysis using two different groups of gene sets:

#### Hypothesis-free

We performed exploratory analyses of a large number of gene sets derived from MsigDB 5.1^38^, including: (i) Gene Ontology (GO) sets of molecular functions (MF), biological processes (BP), and cellular components (CC) (http://www.geneontology.org)^39^; (ii) Reactome database of pathways and reactions in human biology (http://www.reactome.org)^40^; (iii) Kyoto Encyclopedia of Genes and Genomes (KEGG) database (www.genome.jp/kegg)^41^; and (iv) Pathway Interaction Database (PID)^42^. To enhance power, we limited the analysis to gene sets with 100–1000 genes.

#### Hypothesis-driven

In addition, we generated a group of gene sets derived from previous SCZ genetic findings, including: common^9^, rare copy number^11^ and de novo^10^ variants, as well as gene sets associated with rare variants^12^ (fragile X mental retardation protein^43^ and postsynaptic density^44^) and prenatal and postnatal signatures defined based on BrainSpan data (http://www.brainspan.org/).

For both analyses, we only considered genes that were captured by the exon kit. We compared the two distributions of mutation counts between SCZ and controls using the Wilcoxon-rank sum test. The resulting *P*-values were corrected for multiple testing based on the Benjamini and Hochberg method. We also performed bootstrap analysis by random sampling with replacement. We report significant genes sets at FDR ≤0.1 and success rate in bootstrapping (%) >99%. The Haldane–Anscombe correction was applied to calculate the odds ratio when one of the cells has zero value.

### Statistical analysis to compare mutational burden across SCZ cases and controls

We applied linear mixed-effects regression models to test the mutational burden in neuronal and non-neuronal sSNVs among cases with SCZ and controls. Covariates of interest (disease status, cell type, and the interaction of cell type by disease status) and confounds (sex and ancestry) were modeled as fixed effects while donor was modeled as random effects. This statistical model allows for accurate estimates of the means, variances, and significances of each covariate of interest while accounting for the increased uncertainty due donor effects. We tested each covariate of interest for difference from zero, based on a *t*-test using the Satterthwaite approximation on the degrees of freedom. We fit the linear mixed-effects regression models using the function *lmer* from the *lme4* R package (v1.1-17) and the *lmerTest* package (v3.0-1) to perform the Satterthwaite corrected *t*-tests.

### Validation experiments

A number of SNVs identified by WES were selected for validation by Sanger sequencing of cloned PCR products corresponding to the mutated regions and/or by TaqMan-based digital PCR (dPCR).

### Sanger sequencing

PCR primers were designed to amplify the region flanking the nucleotide of interest (Life technologies) (Supplementary Table 2). Following PCR, reactions were resolved on 2% agarose gels and bands of the predicted molecular weight were excised and subjected to gel purification (Qiagen Minelute Gel Extraction Kit—Qiagen cat. #28604). Purified PCR products were sub-cloned in to the zero blunt topo cloning vector (Thermo Fisher Scientific cat. # K280020) according to manufacturer’s instructions. No fewer than 94 colonies from each transformation reaction were then subjected to Sanger sequencing (Genewiz) and the presence or absence of the relevant SNV was determined.

### dPCR

We used TaqMan-based dPCR to validate some of the putative SNVs identified by WES (Supplementary Table 3). Custom TaqMan SNP Genotyping Assays (Life technologies) were performed using the QuantStudio 3D digital PCR 20 Chip Kit v2 (life technologies cat. #A26316) and the QuantStudio 3D digital PCR system.

## Results

### Increased burden of sSNVs in SCZ

**Fig. 1:**
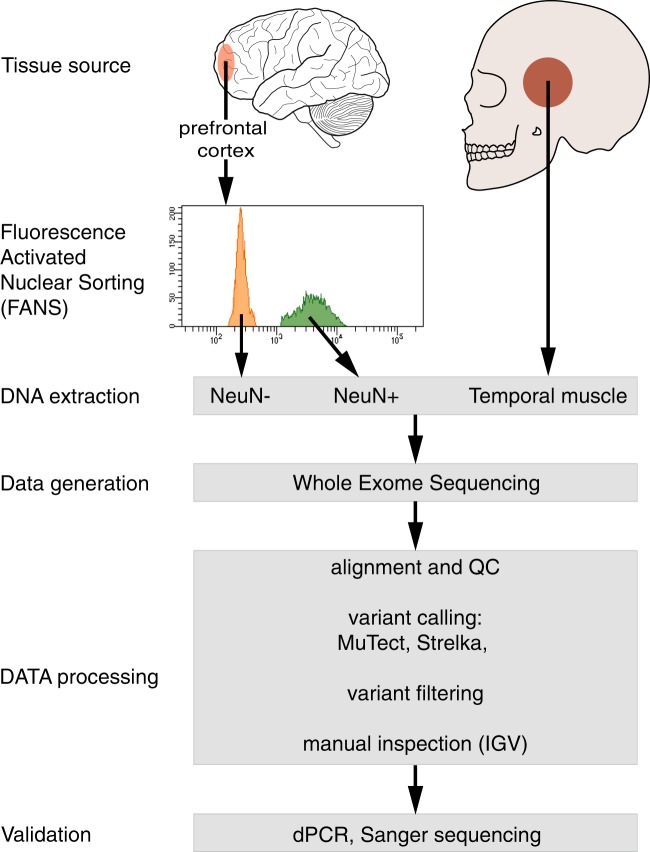
Study design. Genomic DNA isolated from PFC derived neuronal (NeuN+) and non-neuronal (NeuN−) nuclei were subjected to whole-exome sequencing (WES), with DNA from temporal muscle used as an internal reference. Data were aligned to the genome and variants were identified using a combination of methods. Identified sSNVs were validated through direct sequencing of cloned PCR products corresponding to the genomic region of interest and/or by digital PCR (dPCR). QC quality control, IGV Integrative Genomics Viewer

We assessed the presence of sSNVs in neuronal and non-neuronal nuclei isolated by FANS from prefrontal cortex of nine cases with SCZ and 11 controls. From each sample, we obtained WES for DNA extracted from three sources: (i) neuronal (NeuN+) nuclei, (ii) non-neuronal (NeuN−) nuclei, and (iii) a peripheral, non-brain tissue (temporal muscle) (Fig. 1). After alignment, variant calling, and assessment of genetic concordance across tissues labeled as coming from the same donor, we removed one individual due to a biobank sample swap resulting in no matched muscle specimen. The final dataset comprised nine cases with SCZ and 10 controls. For all samples, WES data for neuronal, non-neuronal, and temporal muscle were utilized with the exception of one case with SCZ (individual S2), where the non-neuronal data were excluded due to an implausible excess of somatic variants (Supplemental Materials). The demographics of the final cohort included in this analysis is described in Supplementary Table 1. Cases and controls had similar technical sequencing metrics, including total coverage, proportion of deeply covered targets, and overall proportion of non-reference alleles (Supplementary Table 4).

**Fig. 2:**
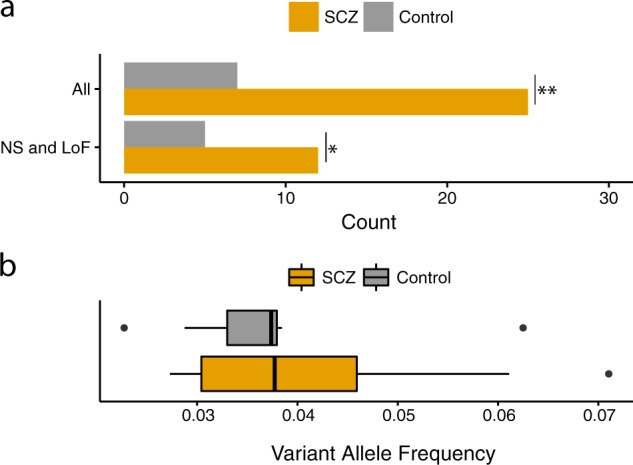
Burden analysis. **a** Count of somatic single-nucleotide variants (sSNVs) in cases with schizophrenia (SCZ) and controls. “All” includes all sSNVs; “NS and LoF” includes the non-synonymous (NS) and loss-of-function (LoF) variants. *P < 0.05; **P < 0.01 for case/control differences estimated based on linear mixed models. **b** Distribution of variant allele frequency of sSNVs in cases with SCZ and controls

To identify sSNVs, for each locus, we compare the fraction of reads harboring the alternate allele (the “variant allele fraction”, or VAF) in two specimens from the same individual: one specimen suspected to contain an sSNV (the “somatic” sample; in this case, neurons or non-neurons) and one specimen not suspected to contain an sSNV (the “reference” sample). sSNVs were identified based on the consensus of two somatic variant callers, selected among other callers as described in the Supplementary Information. A total of 32 sSNVs were in the final call-set, with 25 in cases (12 of which are predicted to be non-synonymous and loss-of-function) and seven in controls (five of which are predicted to be non-synonymous and loss-of-function) (Table 1; Supplementary Figure 4). We detected a significant increase in sSNV burden in cases compared to controls (*P* = 0.0092, linear mixed-effects model) (Fig. 2a and Supplementary Figure 5). No significant effect of cell type (neuron vs non-neuron) or interaction of cell type by case-control status was observed. The rate of non-synonymous (NS) and loss-of-function (LoF) sSNVs was 2.66-fold higher in SCZ compared to controls (SCZ rate = 1.33, control rate = 0.5, *P* = 0.047, linear mixed-effects model; Table 1). All sSNVs were detected in a small proportion of cells. More specifically, on an average, 4% of the reads contained the somatic allele (range 2.2%–7.1%), with no significant differences with regard to this metric in the sSNVs found among cases compared to those found among controls (*P* = 0.67, *t*-test) (Fig. 2b).

**Table 1:** Summary of identified somatic single-nucleotide variants

sSNV	Sample ID	Diagnosis	Cell type	gnomAD allele frequency	Number of reads	Variant allele frequency	Type	Gene	CADD: Raw	CADD: Phred
9:130263483|C>T	C1	Control	Non-Neuronal	0	313	2.88%	Intronic	LRSAM1	0.18	4.52
12:120582181|G>T	C5	Control	Non-Neuronal	0	188	3.72%	Exonic (loss-of-function)	GCN1	6.47	30.00
17:2575935|G>T	C5	Control	Neuronal	0	80	6.25%	Intronic	PAFAH1B1	−0.11	1.68
5:133747485|C>T	C6	Control	Neuronal	1.48E−05	187	3.74%	Exonic (non-synonymous)	CDKN2AIPNL	2.41	18.91
X:25014005|C>T	C6	Control	Neuronal	0	660	2.27%	Exonic (loss-of-function)	POLA1	12.14	38.00
15:42439899|C>T	C10	Control	Neuronal	0	182	3.85%	Exonic (non-synonymous)	PLA2G4F	6.59	31.00
9:2718448|C>T	C10	Control	Non-Neuronal	0	214	3.74%	Exonic (non-synonymous)	KCNV2	5.59	26.50
15:45695252|G>A	S1	SCZ	Neuronal	0	117	4.27%	Exonic (non-synonymous)	SPATA5L1	1.00	10.65
8:6338368|C>T	S1	SCZ	Non-Neuronal	7.45E−05	132	3.79%	Exonic (non-synonymous)	MCPH1	6.53	31.00
1:43850087|C>T	S3	SCZ	Non-Neuronal	5.82E−05	413	3.87%	UTR3	MED8	0.16	4.25
11:120838140|C>T	S3	SCZ	Non-Neuronal	0	173	3.47%	Intronic	GRIK4	−0.01	2.52
4:52779777|C>T	S3	SCZ	Non-Neuronal	0	243	3.29%	UTR3	DCUN1D4	0.94	10.32
X:120183145|C>G	S3	SCZ	Non-Neuronal	0	398	2.76%	Exonic (non-synonymous)	GLUD2	3.92	23.50
X:75650577|C>G	S3	SCZ	Non-Neuronal	0	403	2.73%	Exonic (non-synonymous)	MAGEE1	3.22	22.70
X:77298037|G>A	S3	SCZ	Non-Neuronal	0	117	4.27%	Intronic	ATP7A	−0.13	1.52
9:133267386|G>A	S4	SCZ	Neuronal	0	196	4.59%	Exonic (synonymous)	HMCN2	0.64	8.41
1:228540840|C>T	S7	SCZ	Non-Neuronal	0	242	3.72%	Intronic	OBSCN	0.50	7.44
19:58187980|G>A	S7	SCZ	Non-Neuronal	0	183	7.10%	Intronic	ZSCAN4	0.12	3.87
22:41527619|C>T	S7	SCZ	Non-Neuronal	0	146	4.79%	Exonic (non-synonymous)	EP300	5.93	27.60
4:163032431|G>A	S7	SCZ	Neuronal	1.65E−05	204	4.41%	Exonic (loss-of-function)	FSTL5	10.65	36.00
11:89155084|C>T	S8	SCZ	Non-Neuronal	4.99E−05	180	6.11%	Exonic (synonymous)	NOX4	2.29	18.11
11:89155084|C>T	S8	SCZ	Neuronal	4.99E−05	102	4.90%	Exonic (synonymous)	NOX4	2.29	18.11
12:49360144|G>A	S8	SCZ	Non-Neuronal	1.83E−04	343	4.96%	Exonic (non-synonymous)	WNT10B	7.27	34.00
12:49360144|G>A	S8	SCZ	Neuronal	1.83E−04	409	2.93%	Exonic (non-synonymous)	WNT10B	7.27	34.00
22:32198753|C>T	S8	SCZ	Non-Neuronal	0	263	3.04%	Exonic (non-synonymous)	DEPDC5	7.10	34.00
5:177688699|G>A	S8	SCZ	Non-Neuronal	0	271	2.95%	Intronic	COL23A1	0.17	4.41
16:72166900|G>A	S9	SCZ	Neuronal	0	159	3.77%	Intronic	PMFBP1	1.08	11.11
16:90015951|G>A	S9	SCZ	Neuronal	0	162	3.70%	Exonic (synonymous)	DEF8	0.35	6.14
17:78182086|G>A	S9	SCZ	Neuronal	7.27E−05	273	2.93%	Exonic (non-synonymous)	CARD14	2.84	21.60
19:7566139|G>T	S9	SCZ	Neuronal	0	145	3.45%	Exonic (non-synonymous)	C19orf45	1.00	10.66
22:42373091|G>A	S9	SCZ	Non-Neuronal	0	108	5.56%	Intronic	SEPT3	1.74	14.63
8:30705911|G>T	S9	SCZ	Non-Neuronal	0	431	2.78%	Exonic (non-synonymous)	TEX15	3.62	23.20

### Mutational signature analysis

**Fig. 3:**
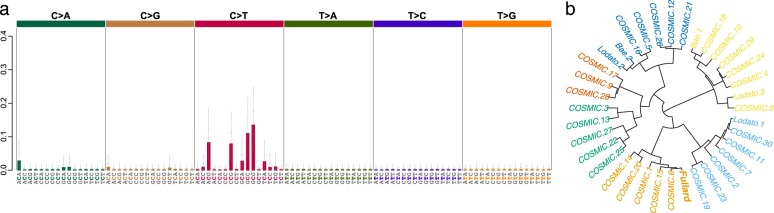
Mutational signature analysis. **a** Barplots showing one mutation signature present in the somatic single-nucleotide variants (sSNVs) in cases with schizophrenia (SCZ) and controls. This signature was comprised mainly of C > T mutations. **b** Hierarchical clustering of mutational signature derived from the current study (Fullard) and three previous studies conducted in single neurons (“Lodato” and “Bae”) and cancer (“COSMIC”). Colors indicate different clusters of mutational signatures

Mutational signature analysis revealed a single signature explaining the mutational spectrum of SCZ and control sSNVs (Fig. 3a). This signature (which we call “Fullard”) was comprised mainly of C > T transitions. We found a larger fraction of C > T transitions in SCZ resulting in a higher transition to transversion (Ti/Tv) ratio of 5.25, compared to 2.5 in controls. We note that there was no case-control difference in Ti/Tv ratio for germline variants called from the same data (average is 2.32 for both cases and controls; Supplementary Table 4). We then compared our results with mutational signatures derived from three studies: two sSNVs studies conducted in single neurons by Lodato et al.^35^ (“Lodato” signatures) and Bae et al.^3^ (“Bae” signatures) and one study in cancer^45^ (“COSMIC” signatures). Unsupervised cluster analysis indicates a different mutational mechanism of “Fullard” with the other 2 brain signatures (Fig. 3b). The “Fullard” signature clusters with COSMIC 1, 6, and 15 signatures, which are related to endogenous mutational process initiated by spontaneous deamination of 5-methylcytosine and defective DNA mismatch repair mechanisms. On the other hand, the “Bae 2” and “Lodato 2” cluster with COSMIC 5 and 16 signatures and resemble a “clock-like signature”, as it was found in nearly all cancer COSMIC samples^45^. “Bae 1” and “Lodato 3” signatures comprise mostly of C > A transversions and they are most closely associated with oxidative DNA damage. The different mutational signatures among our study and the previous studies^3,35^ might be explained by the developmental stage at which the mutations arose. The sSNVs identified in our analysis were present, on average, in 4% of the cells in the specimen, indicating an early incident during development. The “Lodato” and “Bae” signatures derive from single cell sequencing and, therefore, will be detected independent of the developmental stage in which that mutation occurred, i.e. the threshold for detection of a somatic variant at the single cell level is lower than in our study.

### Gene set enrichment analysis

We evaluated enrichment of genes affected by sSNVs in SCZ vs controls using two different groups of gene sets: (i) hypothesis driven, which includes genes previously implicated in SCZ, and (ii) hypothesis free, including gene sets related to biological pathways and molecular functions (see Methods for more details). In the hypothesis-driven set, we found enrichment for two gene sets (de novo mutations and prenatal genes) with SCZ that had odds ratio >5 and survived multiple testing corrections (Table 2). We observed enrichment SCZ sSNVs among the 854 SCZ de novo genes (four SCZ sSNVs in four SCZ de novo genes, zero control sSNVs in SCZ de novo genes; *P* = 0.026, odds ratio = 8.6). Similarly, the 1427 prenatal-biased genes were significantly enriched in SCZ sSNVs (three SCZ sSNVs in three pre-natal genes, zero control sSNVs in pre-natal genes; *P* = 0.026, odds ratio = 6.7). There was no significant enrichment of SCZ sSNVs with hypothesis free gene sets after multiple testing correction (Supplementary Table 5).

**Table 2:** Enrichment analysis using the hypothesis-driven gene sets

Gene set	*P* value	FDR	Success rate in bootstrapping (%)	Gene set size	Overlap with schizophrenia (23 total genes)	Overlap with control (seven total genes)	Odds ratio	Schizophrenia genes
Prenatal brain expression	**0.026**	**0.096**	**100**	**1427**	**3**	**0**	**6.723**	**PMFBP1; OBSCN; ATP7A**
De novo in schizophrenia	**0.026**	**0.096**	**100**	**854**	**4**	**0**	**8.627**	**PMFBP1; TEX15; EP300; OBSCN**
FMRP targets	0.060	0.096	0	776	2	0	4.818	EP300; SEPT3
PGC2-GWAS	0.060	0.096	0	333	2	0	4.818	EP300; SEPT3
Postnatal brain expression	0.060	0.096	0	1396	2	0	4.818	COL23A1; GRIK4
De novo in control	0.145	0.166	–	528	1	0	2.913	EP300
Neuronal proteome:PSD	0.145	0.166	–	659	1	0	2.913	SEPT3
PGC2-CNV	1	1	–	160	0	0	1.008	–

In bold are significant gene sets at FDR ≤0.1 and success rate in bootstrapping (%) >99%. The Haldane–Anscombe correction was applied to calculate the odds ratio

### Validation of sSNVs in using dPCR and Sanger sequencing

**Fig. 4:**
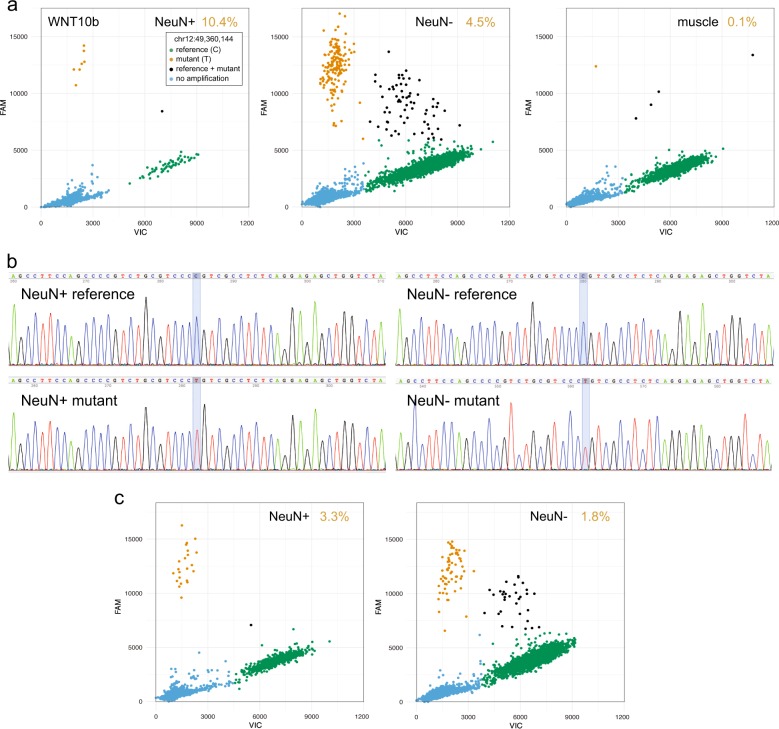
Validation of somatic single-nucleotide variants. Validation of sSNV at chr12:49,360,144 in the gene encoding *WNT10B*. **a** dPCR analysis of sSNV prevalence in DNA extracted from NeuN+ (neuronal) nuclei, NeuN− (non-neuronal) nuclei, and temporal muscle from original dissections. % of mutant (T) allele is indicated. **b** Sanger-sequencing of cloned PCR products containing chr12:49,360,144 (highlighted) from DNA isolated from NeuN+ and NeuN− nuclei. **c** dPCR validation of the sSNV affecting *WNT10B* in an independent dissection

We selected seven NS sSNVs from cases with SCZ for further analysis based on availability of DNA from the same preparation used for WES analysis. Of these, four (in the genes encoding *WNT10B*, *DEPDC5*, *GLUD2*, and *MAGEE1*) were successfully validated by quantitative dPCR (Fig. 4 and Supplementary Figure 6; Supplementary Table 3). To further validate our findings, we sought to confirm a selection of the identified sSNVs by Sanger sequencing of PCR products from multiple clones (clone-seq) designed to span the mutated nucleotide (Supplementary Table 2). We were particularly interested in confirming the mutation identified in *DEPDC5*, as its detection rate by dPCR was low (0.4%) and may have been attributed to the background (Supplementary Figure 6). By clone-seq, the mutations affecting both *DEPDC5* and *MAGEE1* were found in the genome of non-neuronal nuclei, at rate of 1.0% and 3.1%, respectively. As with the dPCR experiment, the *WNT10B* mutation was found in genomic DNA isolated from both neurons (4.3%) and non-neurons (5.3%) (Fig. 4b).

Next, we sought to examine the extent of the clones of mutant cells within the prefrontal cortex. Using independent, adjacent, dissections (0.8 cm posterior to original dissection) from the same individuals, we isolated genomic DNA from neuronal and non-neuronal nuclei, as before. The presence of mutations identified in the original dissections was determined by dPCR. Of the four mutations tested (sSNVs within *WNT10B*, *DEPDC5*, *GLUD2*, and *MAGEE1*), only that affecting *WNT10B* was found in the secondary dissection (Fig. 4c and Supplementary Figure 7) As in the initial experiment, the *WNT10B* mutation was observed in the genome of both neurons and non-neurons, albeit at a lower rate (10.4% and 4.5% vs 3.3% and 1.8%, respectively) (Supplementary Table 6).

Overall, we were able to validate 57% (four out of seven) of sSNVs detected by our bioinformatic analysis using two independent experimental approaches. We failed to detect three of the four validated sSNVs in an additional, adjacent, dissection, indicating that those sSNVs are restricted to discrete clones of cells.

## Discussion

Somatic variation is a major driver of disease, particularly in the context of cancer, and increasing evidence suggests a link between somatic mutation and neurological disorders^5,6^. Single cell approaches have begun to shed light on the contribution of somatic variation to the genetic heterogeneity of the human brain^3,7,35,46–49^. Each neuron in a healthy human brain is estimated to harbor >1000 SNVs^7^. An increasing number of studies provide evidence in support of the notion that somatic variation contributes to defects in brain development and plays a role in neuropsychiatric disease (reviewed in ref. ^6^).

The genetic etiology of SCZ is complex, but a number of recent large-scale studies have made significant progress toward understanding the contribution of common and rare variants to the disease^9–13^. Given the frequency at which monozygotic twins are discordant for SCZ^50^, it is plausible that not all SCZ risk variants are inherited through the germline. Previously, an increased burden of somatic deletions^15^, CNVs^16^, and long interspersed element-1 (L1) retrotransposons^17,18^ have been implicated in SCZ. Thus, we sought to assess the prevalence of sSNVs in postmortem brain specimens isolated from controls and individuals with SCZ. We reasoned that, for a sSNV to contribute to disease, it must meet a number of criteria: (1) the mutation must affect a critical gene but be non-cell lethal (i.e. the cell must be able to propagate despite the variant), (2) cells hosting the mutation must be relevant to the pathophysiology of the disease, and (3) those mutated cells must be sufficiently abundant at the time of death to affect a critical number of cells within the affected tissue. We compared the exome sequence of bulk DNA isolated from neurons, non-neurons, and a peripheral, non-brain tissue (temporal muscle). We identified 32 somatic variants (25 in cases, seven in controls). Of the mutations identified, seven were selected in order to validate our approach, of which four were confirmed.

One such variant was detected in both neurons and non-neurons of a SCZ case and affected the gene encoding *WNT10B*. Wnt signaling plays a critical role in a broad array of cellular process including cell fate determination, polarity, and cell adhesion^51^. With respect to brain function, Wnt signaling has been found to mediate axon guidance, fasciculation, and neural development^52,53^. Numerous studies link aberrant Wnt signaling and SCZ^54–56^. We also identified a SCZ mutation affecting non-neuronal cells in the gene encoding *DEPDC5*, which is an inhibitory component of the TORC1 pathway^57^. An increasing number of studies associate defective *DEPDC5* function with epilepsy^58^ and a somatic mutation has been associated with focal cortical dysplasia^59^. An additional SCZ non-neuron sSNV was identified in the gene encoding the glutamate dehydrogenase, *GLUD2*. *GLUD2* plays a number of important roles during neurotransmission, where it is involved in maintaining synapse integrity^60^, the recycling of glutamate during neurotransmission^61^ and has recently been shown to regulate burst firing of dopaminergic neurons^62^. Although the mutation we detected was restricted to non-neuronal cells, *GLUD2* expression has been shown to be relatively high in astrocytes compared to neurons in the cerebral cortex, and has been hypothesized to facilitate the supportive role played by these cells in neuronal function^63^. Furthermore, activity of *GLUD2* has previously been shown to be elevated in the prefrontal cortex of individuals with SCZ ^64^.

Of the >1000 SNVs to be found in each neuron of the human brain, the vast majority (~80%) have been shown to consist of C > T transitions^7^. In the mouse, this number is ~100 SNVs per neuron, of which ~40% are C > T transitions^65^. C > T transitions have been associated with mutations that arise early in the developing brain^3^. Given that the sSNV affecting *WNT10B* identified in this study was a C > T transition, was found in both neurons and non-neurons, and was detected in two different, adjacent, tissue dissections, we conclude that this mutation occurred relatively early in development and, as such, is more likely to contribute to disease. In addition, these mutational signatures characteristic of early development are supported by our gene set enrichment analysis indicating enrichment with genes active in the prenatal brain (Table 2).

Our validation rate (57%) is notably lower than those reported in cancer studies^32^. The allelic fractions in cancer biopsies, however, are typically higher (~20%) than those seen in our data. Even at the deep coverage used in this study (250× in the brain tissue), calling somatic variants when the fraction of cells harboring the variant is low (e.g. ~5%) relies on detection of a small number of reads harboring the variant allele. Future study designs might mitigate this issue through deeper sequencing and, as more data become available, through the utilization of computational approaches to assist in the identification of false positives^66^.

In this study, we chose to focus on exomes, as the potential impact of variants found therein is more readily discernible. Future studies should explore the somatic variation by applying whole-genome sequencing approaches, and include exploration of other variants such as L1 retrotransposons and structural variation. As our study included a relatively small number of samples, we are insufficiently powered to conclude that the greater number of non-synonymous somatic variants found in SCZ brain tissue is a characteristic of the disease. In addition, validation experiments using animal models are required to determine the phenotypic effects of identified mutations. One approach might be to assess the effect of suppression (or overexpression) of homologous candidate genes in Zebra fish, similar to experiments used in a previous study to determine the impact of modulation of SCZ candidate gene expression on neuroanatomy^23^. An additional approach could employ transposon or CRISPR-Cas9-mediated mutagenesis of brain organoids to determine the structure/function effects of specific somatic mutations^67^.

Despite the limitations of our study, we provide a methodology toward assessing the role played by somatic mutation in SCZ. Additional studies in twins discordant for SCZ, or including larger number of samples (such as those proposed by The Brain Somatic Mosaicism Network^6^), may lead to the identification of previously unknown disease-associated genes or pathways and, in turn, to the discovery of novel therapeutic targets.

## Supplementary information

Supplement

Supplementary Figure 4

Supplementary Table 1

Supplementary Table 2

Supplementary Table 3

Supplementary Table 4

Supplementary Table 5

Supplementary Table 6
