# Supplementary material for: Assessment of somatic single-nucleotide variation in brain tissue of cases with schizophrenia
Source: Transl Psychiatry. 2019 Jan 17;9:21. doi: 10.1038/s41398-018-0342-0 (PMC6336839; doi:10.1038/s41398-018-0342-0)
Supplement: Supplementary file 1 — Supplement [file 41398_2018_342_MOESM1_ESM.docx]

# Supplementary Information

## Comparison of 6 somatic single-nucleotide variant (sSNV) callers

There is currently no gold standard algorithm for calling sSNVs from bulk next-generation sequencing data. We therefore evaluated 6 sSNV callers in order to identify the combination of callers that would allow us to maximize the number of true positive calls. Binary sequence alignment/map (BAM) formatted files containing aligned sequence reads from whole-exome sequencing (WES) of 2 tissues from the same individual were used as input for each caller. The 6 callers are based on different mathematical models but are rooted on the same overarching concept. In brief, for a given locus this consists of comparing the fraction of reads harboring the alternate allele (the “variant allele fraction”, or VAF) in two specimens from the same individual: 1 specimen suspected to contain a sSNV (the “somatic” sample) and 1 specimen not suspected to contain a sSNV (the “reference” sample). As our goal was to identify sSNVs in the brain, the neuronal and non-neuronal exomes were input as the somatic samples. Each caller was then run twice for that sample: once using the temporal muscle tissue as the reference and one using the other brain cell type as the reference. The calling parameters and initial filters applied for each caller were as follows:

- MuTect^8^ (v1.1.6) was run using the default somatic calling parameters with 3 exceptions: the baqGapOpenPenalty was set to 30; the interval padding was set to 50 base pairs; contamination estimates were derived for each specimen using VerifyBamID. The resulting variant call file was then filtered for bi-allelic sSNVs passing the algorithm’s built-in benchmarks.
- Varscan^11^ (v2.3.6) was run using the default parameters for the “somatic” subcommand. Input values for specimen purity, rather than the default of 1, were derived from the contamination estimates of VerifyBamID. The resulting variant call set was then filtered using the "somaticFilter" subcommand of the Varscan pipeline. This step keeps sSNVs that meet all of the following criteria: coverage > 10, > 4 reads harboring the alternate allele, VAF > 0.20, no indels in close proximity, and VAF statistically different between the somatic and reference samples (Fisher's Exact Test p-value < 0.05). Since Varscan reports genotypes as IUPAC ambiguity codes, a small number of sSNVs were removed for having an IUPAC ambiguity code that was incongruent with the reference and alternate alleles at that position (e.g., an IUPAC code of M at a site where the reference and alternate alleles are C and G, respectively). All other bi-allelic sSNVs were retained.
- Strelka^9^ (v1.0.14), EBCall^12^ (v2), Lancet^13^ (v1.0.0) and MultiSNV^14^ (v2.0) were run using the default somatic calling parameters. Only those bi-allelic sSNVs passing the algorithm’s built-in filters were kept. Variants called somatic were retained regardless of the genotype of the “normal” sample.

To assess the performance of these callers, we first merged their outputs into a single set that was filtered to keep only bi-allelic sSNVs aligned to the autosomal and sex chromosomes. Initial checks of this unfiltered call set revealed one non-neuronal sample that was an extreme outlier for the number of sSNVs called (see below). This sample was removed from all analyses, leaving 143,376 putative sSNVs in the unfiltered set of calls. As has been noted by others^15^, consensus across callers was poor, with 91.4% of calls being identified by only 1 of the 6 callers (**Supplementary Figure 1**). A component of this discordance results from the default parameters used by each algorithm, such as the minimum VAF for a site to be considered somatic (**Supplementary Figure 2*)***. Significant differences across callers was also observed with respect to transition/transversion ratio (chi-squared p-value < 2.2 x 10^-16^), number of sSNVs per chromosome (chi-squared p-value < 2.2 x 10^-16^) and relative position of sSNVs across chromosomes (ks.test p-value < 1.0 x 10^-10^ for all pairwise combinations of the 6 callers evaluated).

In light of the large differences between callers, we sought to utilize the combination of callers whose intersect was most likely to yield true positive sSNV calls. To estimate this, we derived two metrics for each caller from the initial set of sSNVs passing the caller’s built-in filters: the fraction of sSNVs by >=1 other caller, and fraction surviving a conservative in-house filtering pipeline that included manual inspection with IGV (see below). Rates of validation by other callers were significantly lower in Varscan and MultiSNV (0.11 and 0.13, respectively) than the other 4 callers (rates ranging from 0.21-0.34; t-test p-value = 0.02). Low rates of validation by the in-house filtering pipeline were seen for all callers (ranging from 0 in Varscan to 0.009 in MuTect); we chose the top 2 callers with regard to this metric (MuTect and Strelka) as the combination required to be retained in the final somatic SNV call set.

## Assessing excess somatic SNV calls across individuals

Prior to employing our in-house filtering pipeline, we assessed the number of putative sSNV calls made per sample by MuTect and Strelka in order to identify potential outliers. A total of 4 specimens (2 neuronal, 2 non-neuronal) from 3 individuals were observed to harbor excess sSNVs compared to the rest of the cohort. For 3 of these specimens, the excess number of calls was the result of low-level contamination (see below). For the other sample (a non-neuronal specimen), several potential explanations of the excess were investigated including low-level contamination, oxidation artifacts, and batch effects (e.g., library, flow cell lane). Extensive investigations were unable to identify the source of the artifact and this specimen was therefore removed from all analyses (data not shown). In the final analysis set of 32 variants, no individual outliers were identified with respect to individual or cell type (**Supplementary Figure 5**).

## False positive calls from low-level contamination

A source of false positive sSNV calls in next-generation sequencing experiments is low-level contamination, whereby a subset of the reads at any given locus are from the DNA of other individuals. To illustrate why low-level contamination requires greater consideration in calling sSNVs compared to germline SNVs, let us consider a hypothetical scenario where a locus has been sequenced to a depth of 100 in an individual who is truly homozygous for the reference allele. In the absence of contamination, the expectation is that 100% of reads covering this locus will contain the reference allele. In the presence of 10% contamination by DNA from an individual who is heterozygous at this locus, approximately 5% of the reads will contain the alternate allele. Germline SNV callers, which are looking for evidence that roughly half of the reads harbor the alternate allele (i.e., evidence of heterozygosity) would likely call this site as homozygous reference despite the contamination. In contrast, sSNV callers such as those used in the current study would likely identify this as a sSNV so long as the reference tissue was not also contaminated. To combat this known source of artifact, MuTect incorporates contamination estimates for the somatic sample into its model. Though we ran MuTect in decontamination mode using contamination estimates derived from VerifyBamID^5^, we nonetheless observed a significant relationship between the number of variants called and the estimated level of contamination (**Supplementary Figure 3a**). Of the 4 exomes found to have excess sSNVs prior to our in-house filtering pipeline (see above), 3 were from the 2 individuals in the dataset with the highest contamination estimates (muscle of individual S5, 3.7% contamination; neuron of individual C9, 1.9% contamination). These two samples were not found to be outliers with respect to any other quality control metrics considered (**Supplementary Table 4**). There was no evidence of either increased identity-by-descent relatedness between these samples and their multiplex pairs or higher rates of discordant genotype calls between these specimens and microarray data from the same individuals (data not shown), as has been found previously in studies with low-level contamination of WES data^7^.

To remove false positive sSNVs resulting from low-level contamination we sought to identify features that could be used to differentiate them from potentially true positive calls. Since most loci in the human genome are invariant^16^, we hypothesized that false positive sSNVs resulting from low-level contamination would primarily occur at sites known to be polymorphic within the population. Barring the existence of a biological mechanism by which sites more likely to vary between individuals in a population are also more likely to vary between cells in an individual, somatic mutations should be enriched for occurring at sites where non-reference alleles have either never been observed or observed in rare instances. If one were to observe high rates of somatic variants at known polymorphic sites, then, it would be suggestive of a technical artifact. For each putative somatic site, a consensus minor allele frequency (MAF) was determined by choosing the larger of the two MAF values observed for the site in the ExAC^17^ and 1000 genomes^16^ datasets. Consistent with the hypothesis that low-level contamination would result in excess somatic SNVs at sites of common variation, we found that the excess somatic calls in the two samples with the highest contamination estimates were at sites with high population MAF. In contrast, the exome with the greatest number of somatic calls (non-neurons of Individual S2; see above), did not demonstrate this characteristic pattern, nor did the exome with the lowest contamination estimate in the dataset (neurons of individual C2; **Supplementary Figure 3b**). The false positive calls due to contamination are of two types: those where reference alleles contaminate a site where the somatic specimen is truly homozygous for the alternate allele, and those where alternate alleles contaminate a site where the somatic specimen is truly homozygous for the reference allele (**Supplementary Figure 3­b)**. By using a MAF threshold of 0.001, the great majority of these false positive associations can therefore be removed and these samples may still be included in the final analysis set.

## Unified allele counts

Since different callers return different allele counts for the same variant based on the read filters applied to input BAM files, in addition to considering the read counts output by each caller we utilized unified allele counts (UACs) in this steps of our in-house filtering pipeline that utilized information regarding the number or fraction of reads harboring the alternate allele. Computation of UACs was performed using the bam­readcount tool (<https://github.com/genome/bam-readcount>), which for each variant generates 4 counts that are independent of the variant caller: number of reference and alternate alleles in the somatic sample, and number of reference and alternate alleles in the reference samples. From these counts were calculated the VAF metrics presented throughout this report.

## Manual inspection procedure

The final step of our in-house sSNV filtering pipeline consisted of manual inspection of aligned reads at putative somatic sites using IGV. The manual inspection procedure we employed was based on anecdotal evidence from our group and others with experience performing sSNV calling. In general, sSNVs were marked to be removed if from the IGV inspection of aligned reads there was evidence of any of the following: nearby structural variants, excessive soft-clipping on alternate reads relative to non-alternate reads (in self or mate, where the soft-clipped sequence does not match adapter sequence used in the experiment), excessive mismatches on alternate reads relative to non-alternate reads (on self or mate), nearby variant alleles in or out of phase with the putative sSNV, excessive germline SNVs in the 1000 base pair region surrounding the putative sSNV, the presence of mapQ0 reads or reads with other evidence of low quality (i.e., mapping qualities less than 60 or base qualities less than 20), evidence for true heterozygosity at the putative sSNV site, evidence of strand bias, reads containing the alternate allele in the reference tissue, or evidence the sSNV site mapped to an intergenic region.

## References

1. Linderman, M. D. *et al.* Analytical validation of whole exome and whole genome sequencing for clinical applications. *BMC Med. Genomics* **7,** 20 (2014).

2. Li, H. & Durbin, R. Fast and accurate short read alignment with Burrows-Wheeler transform. *Bioinformatics* **25,** 1754–1760 (2009).

3. DePristo, M. A. *et al.* A framework for variation discovery and genotyping using next-generation DNA sequencing data. *Nat. Genet.* **43,** 491–8 (2011).

4. McKenna, A. *et al.* The genome analysis toolkit: A MapReduce framework for analyzing next-generation DNA sequencing data. *Genome Res.* **20,** 1297–1303 (2010).

5. Jun, G. *et al.* Detecting and Estimating Contamination of Human DNA Samples in Sequencing and Array-Based Genotype Data. *Am. J. Hum. Genet.* **91,** 839–848 (2012).

6. Picard: A set of command line tools (in Java) for manipulating high-throughput sequencing (HTS) data and formats such as SAM/BAM/CRAM and VCF. (2017). at <http://broadinstitute.github.io/picard/>

7. Purcell, S. M. *et al.* A polygenic burden of rare disruptive mutations in schizophrenia. *Nature* **506,** 185–190 (2014).

8. Cibulskis, K. *et al.* Sensitive detection of somatic point mutations in impure and heterogeneous cancer samples. *Nat. Biotechnol.* **31,** 213–219 (2013).

9. Saunders, C. T. *et al.* Strelka: accurate somatic small-variant calling from sequenced tumor–normal sample pairs. *Bioinformatics* **28,** 1811–1817 (2012).

10. Robinson, J. T. *et al.* Integrative genomics viewer. *Nat. Biotechnol.* **29,** 24–6 (2011).

11. Koboldt, D. C. *et al.* VarScan 2: somatic mutation and copy number alteration discovery in cancer by exome sequencing. *Genome Res.* **22,** 568–76 (2012).

12. Shiraishi, Y. *et al.* An empirical Bayesian framework for somatic mutation detection from cancer genome sequencing data. *Nucleic Acids Res.* **41,** e89–e89 (2013).

13. Narzisi, G. *et al.* Lancet: genome-wide somatic variant calling using localized colored DeBruijn graphs. *bioRxiv* 196311 (2017). doi:10.1101/196311

14. Josephidou, M., Lynch, A. G. & Tavaré, S. multiSNV: a probabilistic approach for improving detection of somatic point mutations from multiple related tumour samples. *Nucleic Acids Res.* **43,** e61–e61 (2015).

15. Cai, L., Yuan, W., Zhang, Z., He, L. & Chou, K.-C. In-depth comparison of somatic point mutation callers based on different tumor next-generation sequencing depth data. *Sci. Rep.* **6,** 36540 (2016).

16. Gibbs, R. A. *et al.* A global reference for human genetic variation. *Nature* **526,** 68–74 (2015).

17. Lek, M. *et al.* Analysis of protein-coding genetic variation in 60,706 humans. *Nature* **536,** 285–291 (2016).

**Supplementary Figures**

**Supplementary Figure 1***.* Number of somatic single-nucleotide variants (sSNVs) passing built-in filters for each of 6 sSNV callers evaluated. The intersection of the callers is visualized as an UpsetR plot, which is akin to a Venn diagram in a matrix layout. Each column of the matrix represents the intersection of the subset of the callers indicated by the dark circles, and the vertical black bar indicates the size of the intersection. The blue horizontal bars to the left of the caller labels indicate the number of sSNVs identified for the corresponding caller.


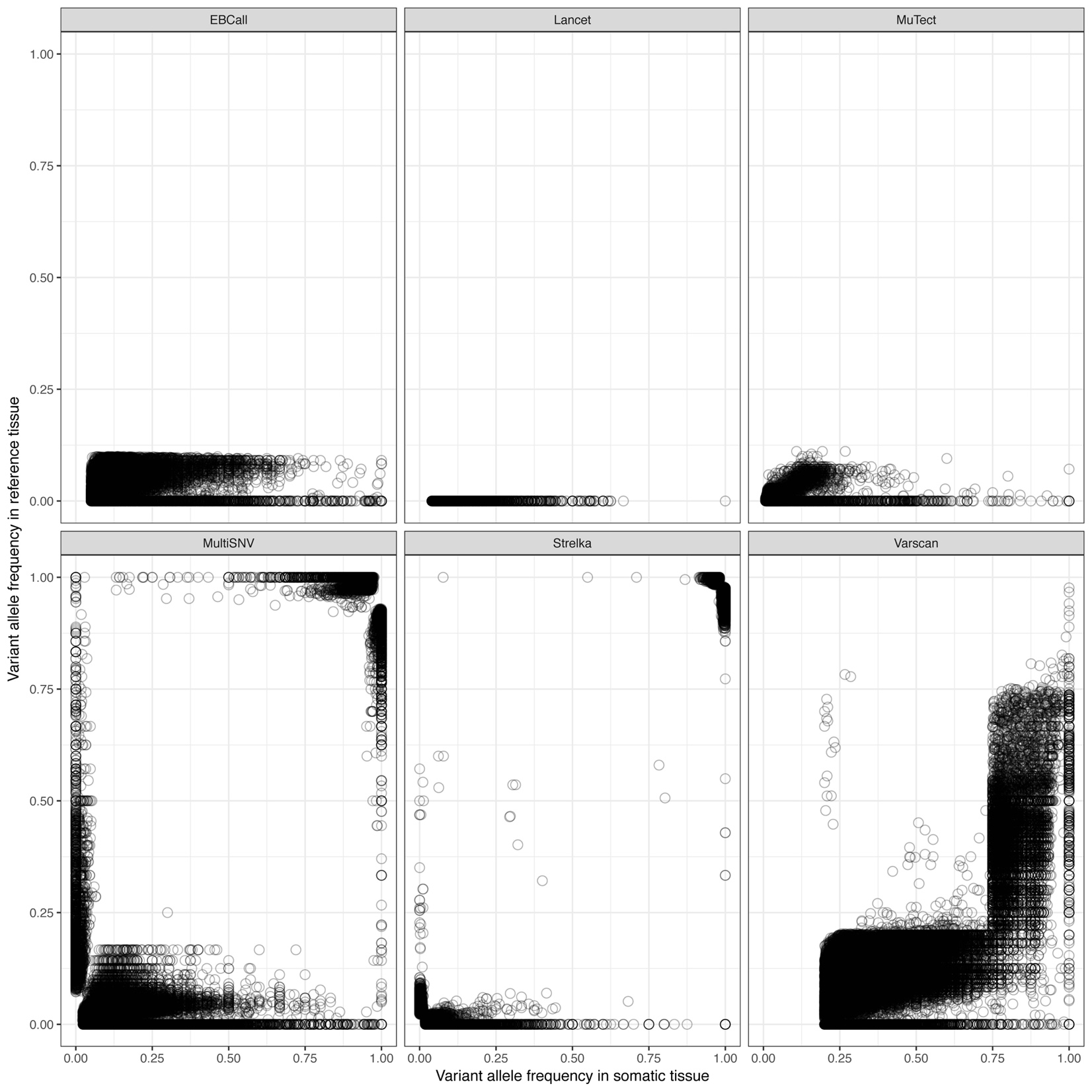


**Supplementary Figure 2***.* Variant allele fraction (VAF) in somatic and reference tissue for all somatic single-nucleotide (sSNV) calls. For each of the 6 callers evaluated, each point in the corresponding plot is a sSNV that passed the software’s built-in filters. The x-axis and y-axis indicate the fraction of reads harboring the alternate allele in the somatic and reference samples, respectively. Default settings of the various algorithms result in the distinct features for each plot. For instance, Varscan requires a minimum VAF of 20% in the somatic sample to call a sSNV, whereas this threshold is lower for the other 5 callers.


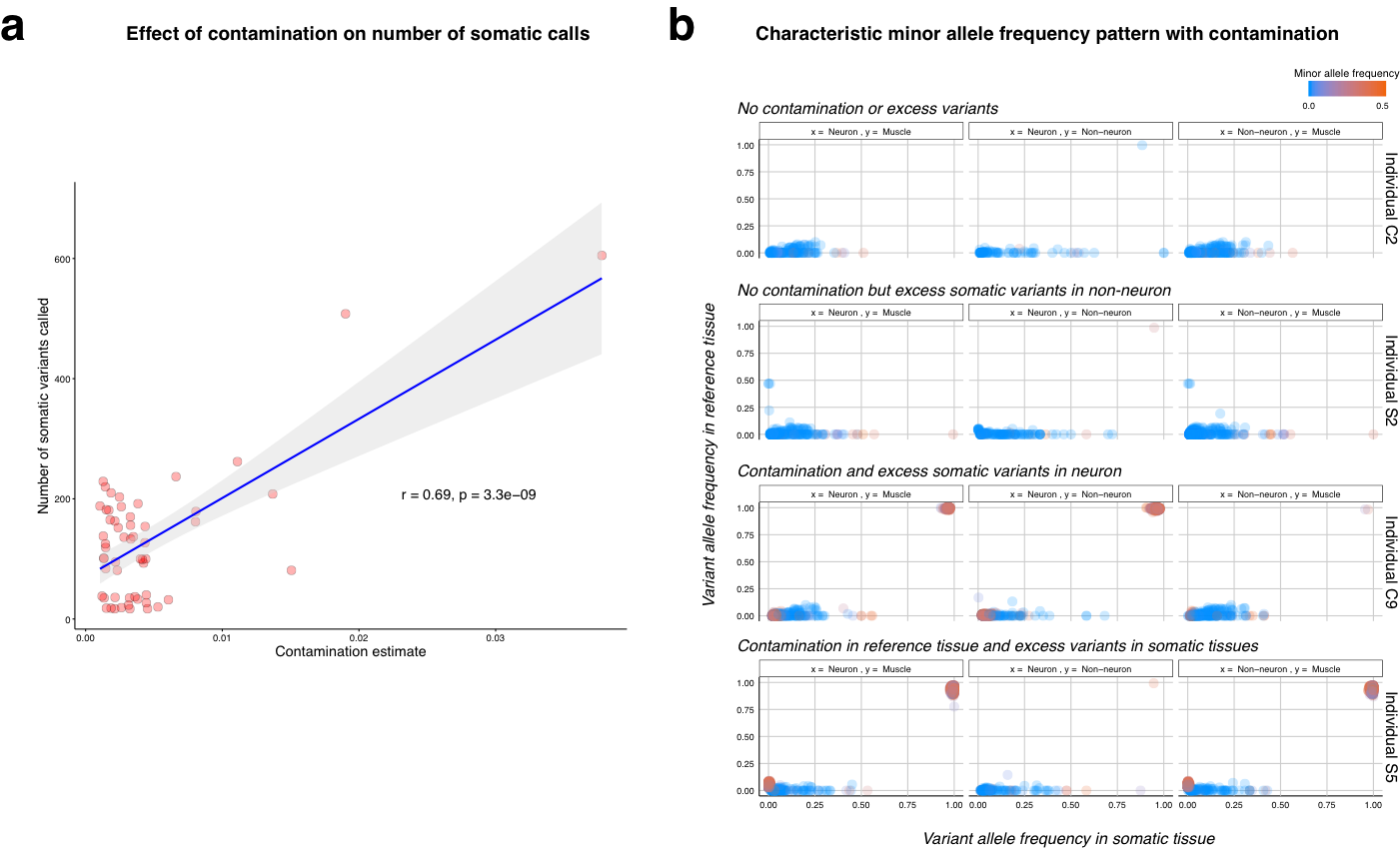


**Supplementary Figure 3***.* Contamination leads to excess somatic single-nucleotide variant (sSNV) calls at commonly polymorphic sites. a) For each exome, the x-axis is the contamination estimate derived from VerifyBamID and the y-axis is the number of sSNVs called by MuTect that passed the software’s built-in filters. Shown are the Pearson’s coefficient (r) and p-value (p) for correlation between the two variables plotted. To avoid confounding, this figure excludes the 1 extreme outlier for number of sSNV calls (Individual S2, non-neurons). b) Depending on the contamination sample, low-level contamination results in a characteristic pattern of false positive sSNV calls with respect to the population minor allele frequency (MAF) and the fraction of reads containing the alternate allele (the variant allele fraction, or VAF). This is illustrated here in 4 series of “fireball” plots. For each plot, the x-axis and y-axis depict the VAF in the somatic and reference tissues, respectively. Points are colored along a spectrum from blue to red according to MAF, with red indicating higher values. Each series contains 3 plots that differ according to the tissues input as somatic and reference in the calling algorithm. In the top two series (Individuals C2 and S2, respectively), no contamination is present, and there is not an excess of somatic calls with high MAF even when there is extreme excess of sSNV calls (as in non-neuron exome of Individual S2). When the somatic tissue is contaminated, as is the case for neuron exome in the 3rd series (Individual C9), excess sSNV calls with high MAF are seen regardless of the reference tissue that is used for comparison. When the contamination is in the reference tissue, as is the case for muscle exome in the 4th series (Individual S5), the excess somatic calls with high MAF are observed regardless of the somatic tissue to which it is compared.

| **See supplemental file “IGV”**  **for Supplementary Figure 4** |
| --- |

**Supplementary Figure 4.**  Presented are 30 plots made using the Integrative Genomics Viewer (IGV) for the final set of filtered somatic single-nucleotide variants (sSNVs). In each plot, the top panel is an ideogram of the chromosome with a vertical red bar indicating the position of the region harboring the sSNV and the range of positions for which alignments are presented. Alignments are shown for all three tissues from the individual in which the sSNV was identified, and the name of the tissue containing the sSNV is shaded. Reference alleles appear gray and non-reference alleles appear colored according the nucleotide (green – adenine, red – thymine, brown – guanine, blue – cytosine). Reads are sorted by the allele at the putative sSNV site. Coverage tracks are shown for each tissue, and sites harboring >=3% alternate reads are highlighted according to the alternate allele using the same color codes described above. The number range in the top left corner of the coverage track indicates the minimum and maximum depths for the range depicted. Tracks showing the annotated RefSeq and codon positions are also included either above or below the alignments.

**Supplementary Figure 5.** Number of somatic single-nucleotide variants (sSNVs) called per individual. For each individual (y-axis) is shown the number of sSNVs in the final analysis set of 32 variants (x-axis). Individuals are colored according to phenotype (cases in yellow, controls in black), and bars are shaded according to whether all variants are counted (dark shade) or just the non-synonymous (NS) and loss-of-function (LoF) variants are counted (light shade).

**Supplementary Figure 6.** Validation of sSNV in the genes encoding *DEPDC5* (**a,b**), *MAGEE1* (**c,d**) *and GLUD2* (**e**). (**a, c, e**) dPCR analysis of sSNV prevalence in DNA extracted from NeuN+ (neuronal) nuclei, NeuN- (non-neuronal) nuclei and temporal muscle from original dissections. % of mutant (T) allele is indicated. (**b, d**) Sanger-sequencing of cloned PCR products from DNA isolated from NeuN+ and NeuN- nuclei for the variants identified in *DEPDC5* and *MAGEE1*. The variant affecting *GLUD2* was not verified by Sanger-sequencing.

**­**

**Supplementary Figure 7.** dPCR analysis of sSNV prevalence in DNA extracted from NeuN+ (neuronal) nuclei, NeuN- (non-neuronal) nuclei from secondary dissections. % of mutant (T) allele is indicated. (**a**) *DEPDC5*, (**b**) *MAGEE1 and* (**c**) *GLUD2.*

**Supplementary Tables**

**Supplementary Table 1:** Sample demographics

**Supplementary Table 2:** Oligonucleotides used for validation of identified SNVs by Sanger sequencing

**Supplementary Table 3:** Oligonucleotides and probes used for validation of identified SNVs by digital PCR (dPCR)

**Supplementary Table 4:** Whole exome sequencing (WES) data metrics

**Supplementary Table 5:** Hypothesis free gene sets following multiple testing correction

**Supplementary Table 6:** dPCR validation data
