# Supplementary figures and images for: Assessment of somatic single-nucleotide variation in brain tissue of cases with schizophrenia
Source: Transl Psychiatry. 2019 Jan 17;9:21. doi: 10.1038/s41398-018-0342-0 (PMC6336839; doi:10.1038/s41398-018-0342-0)

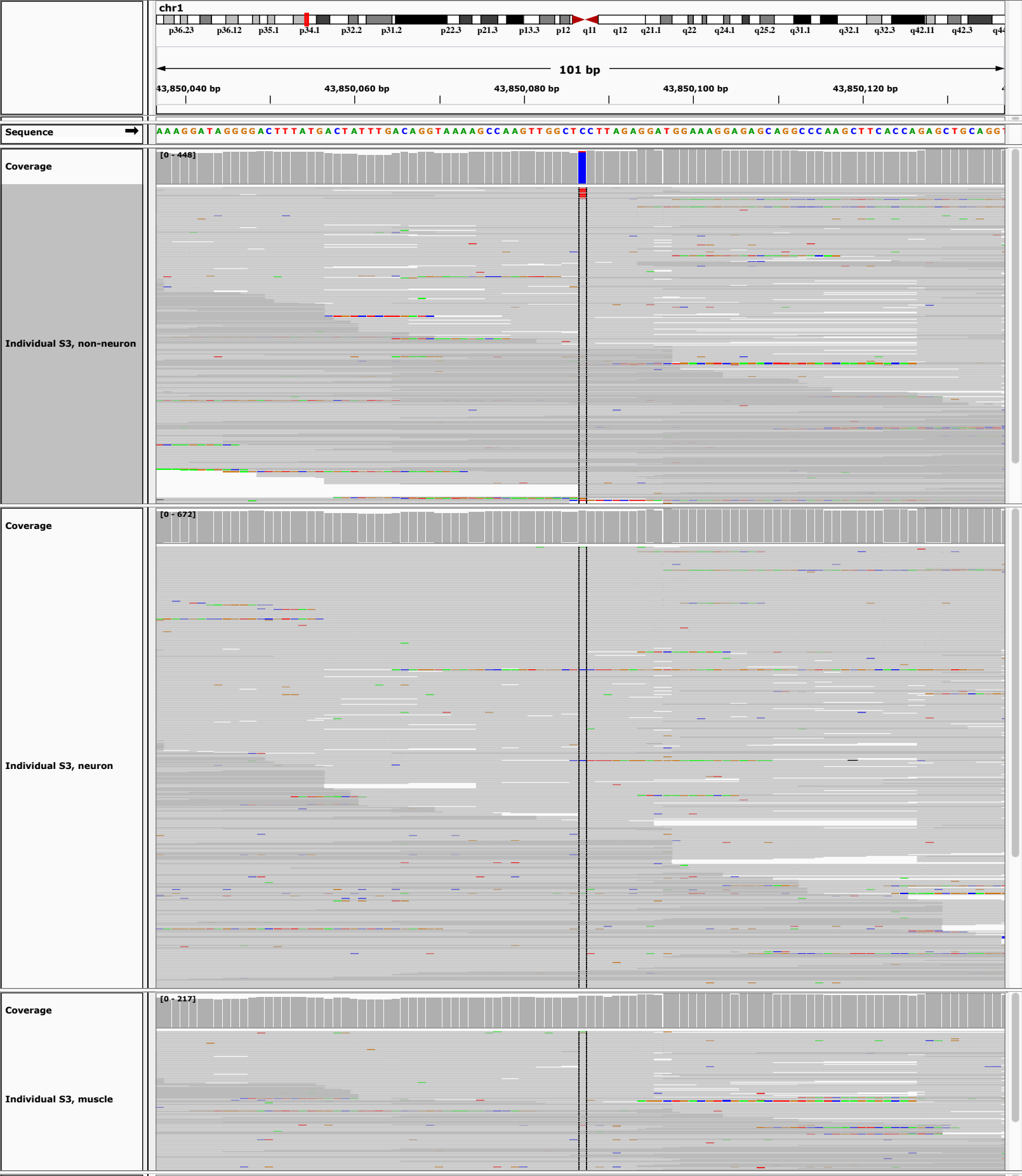

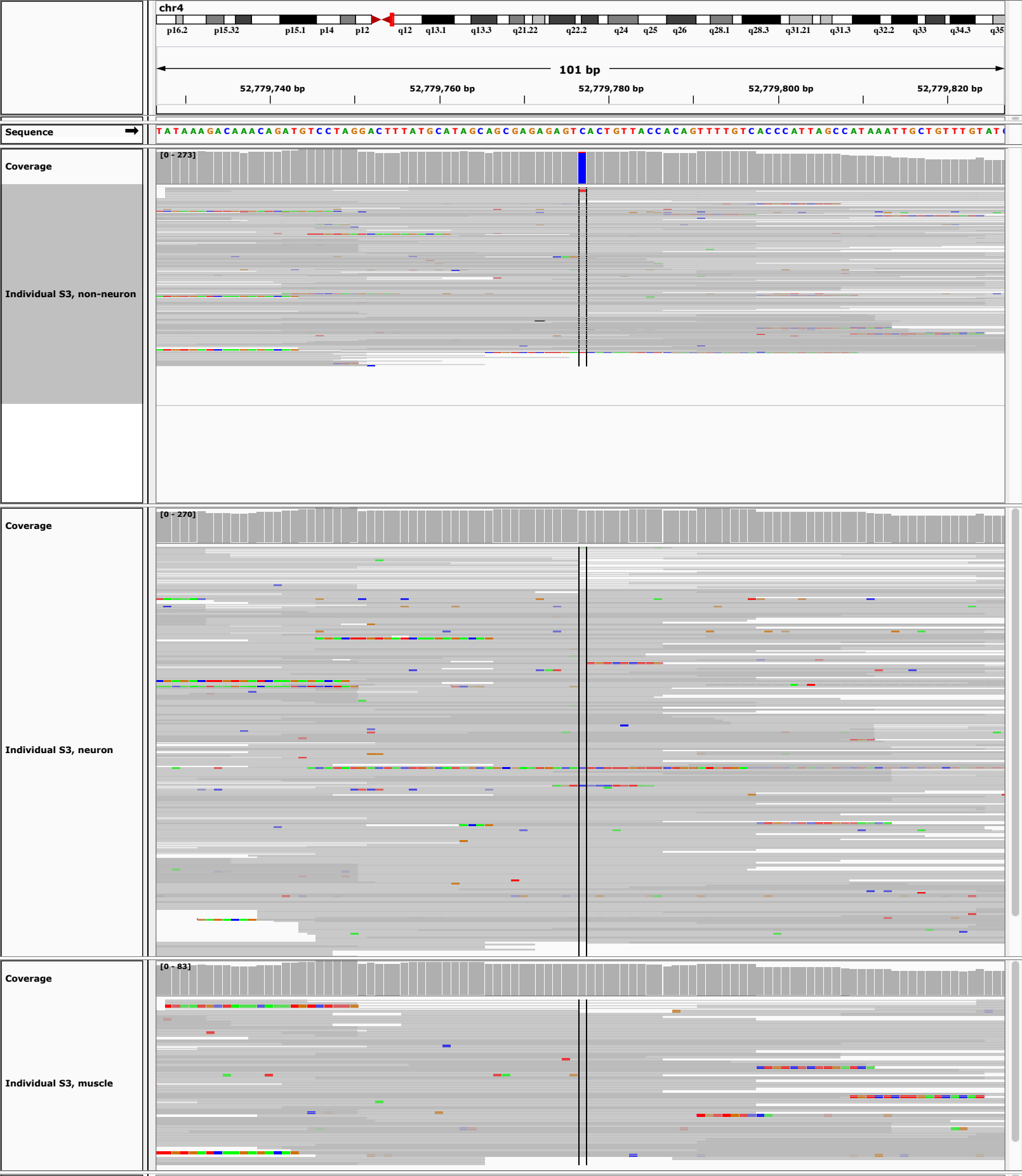

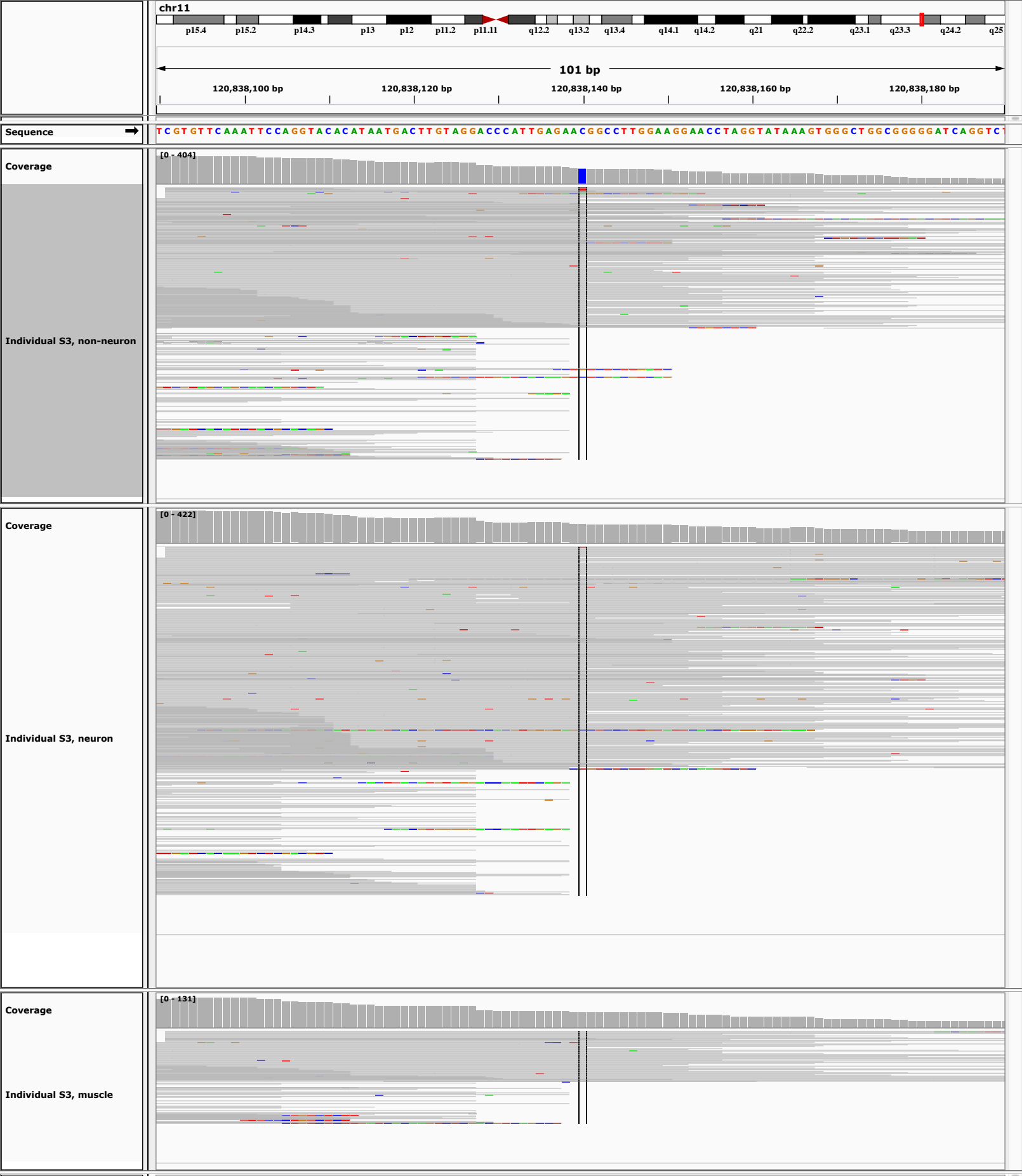

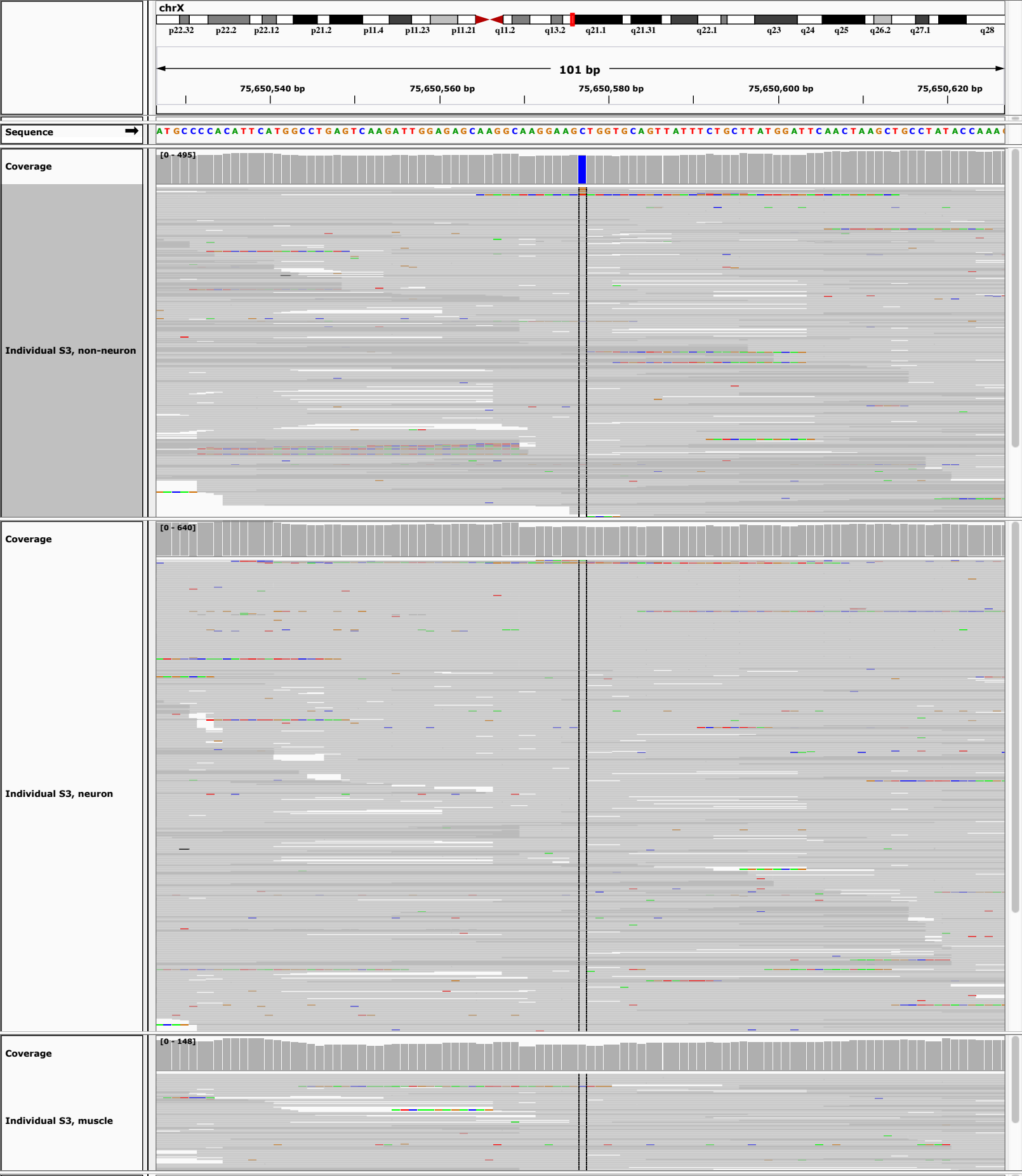

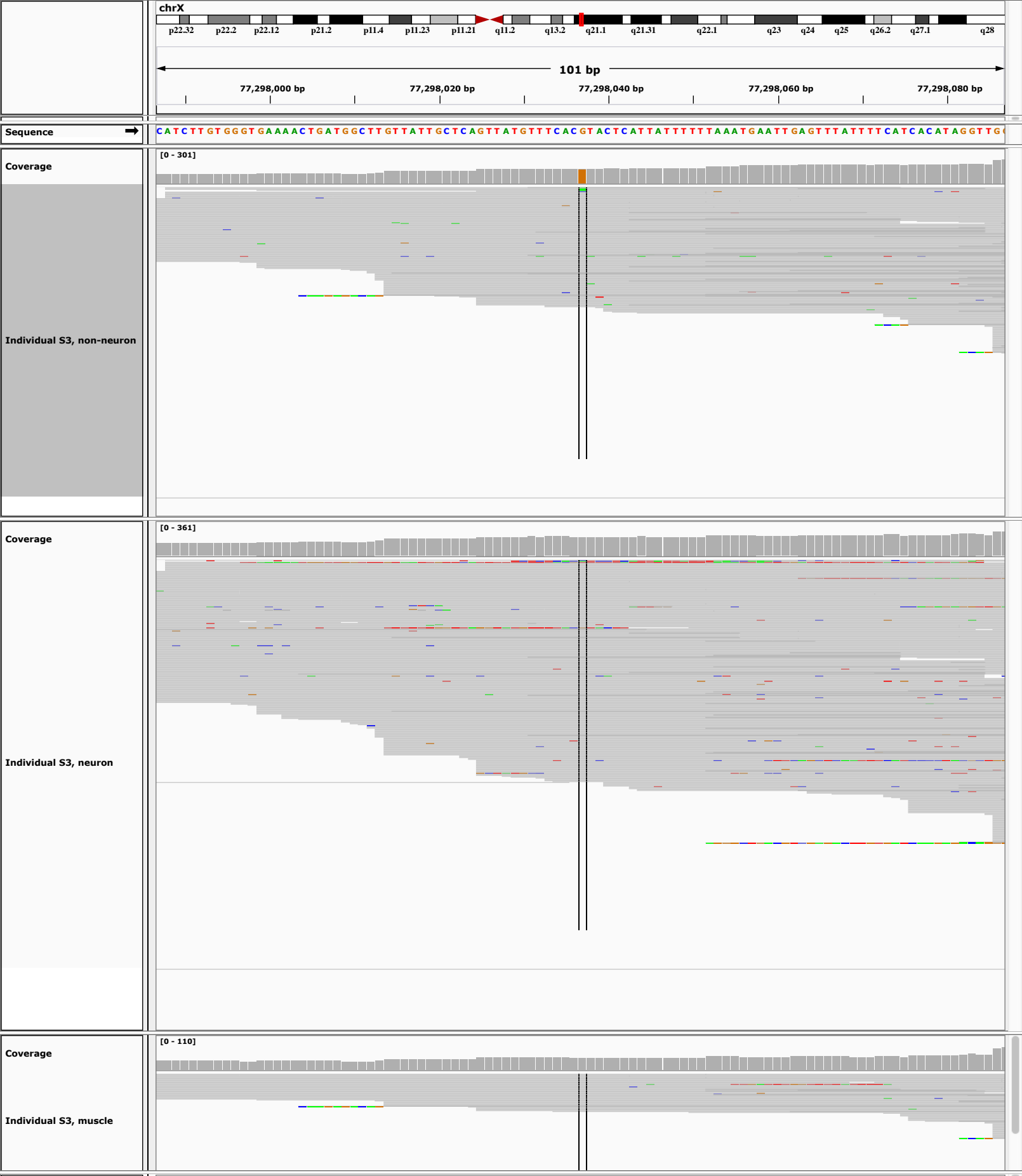

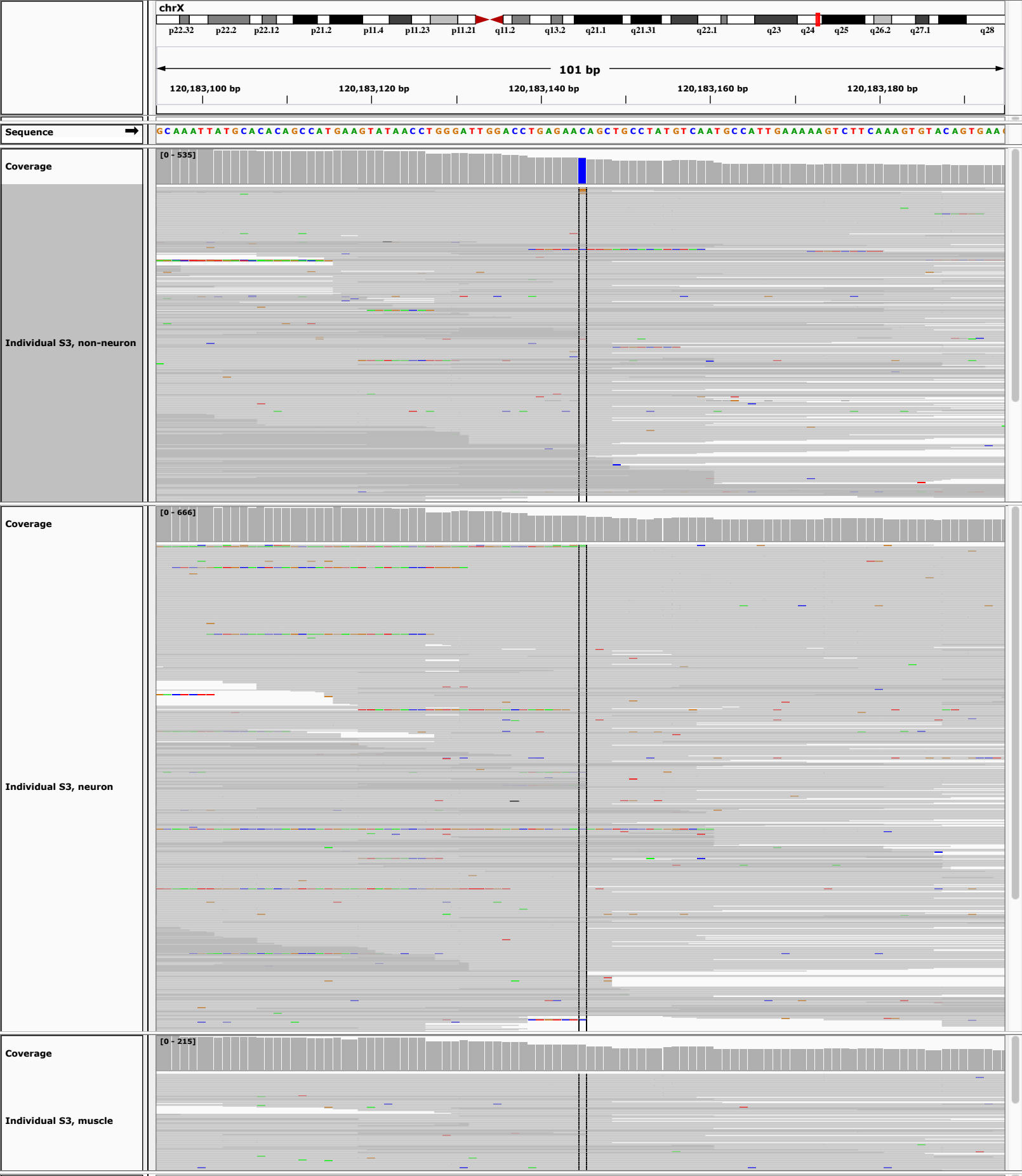

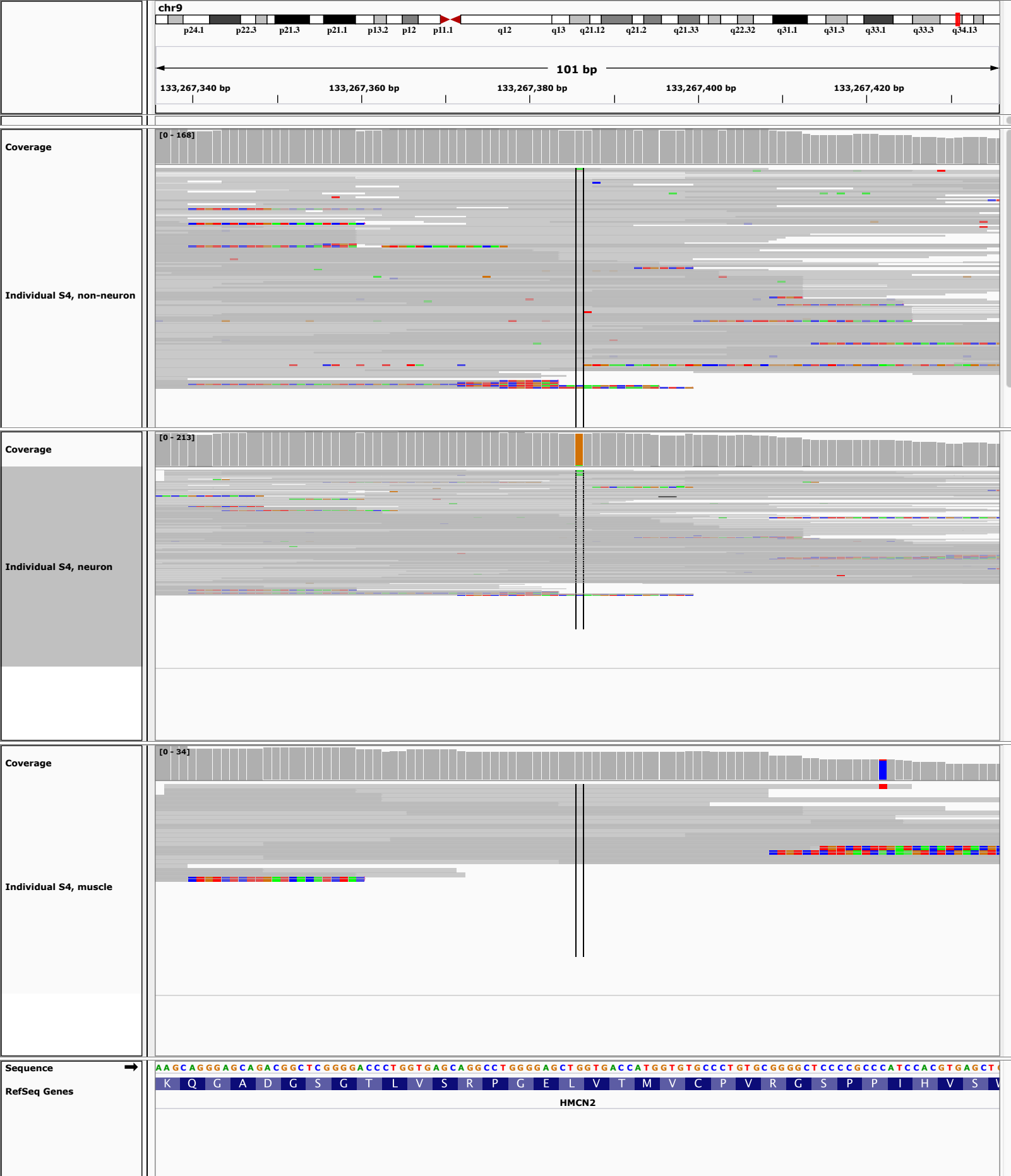

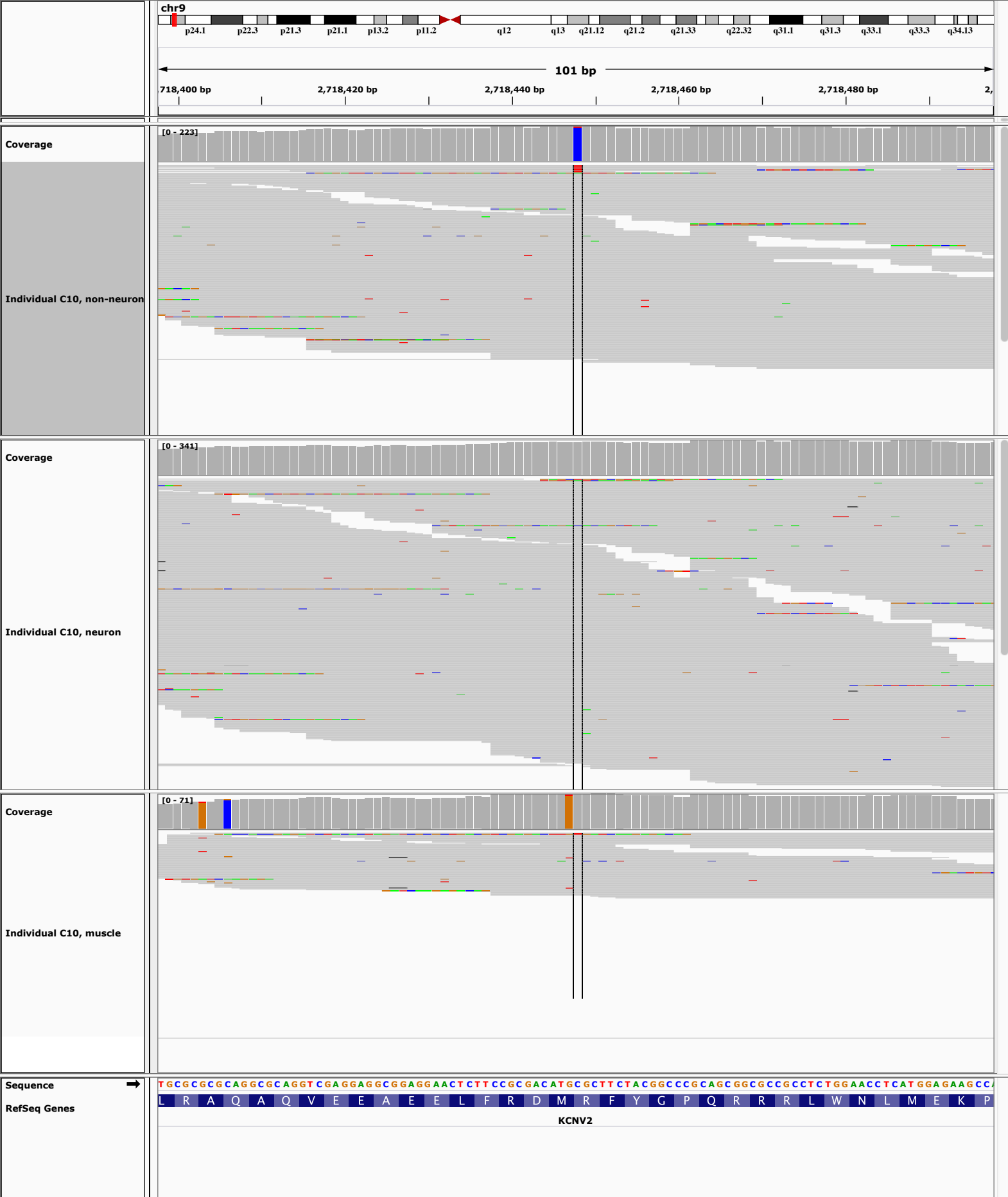

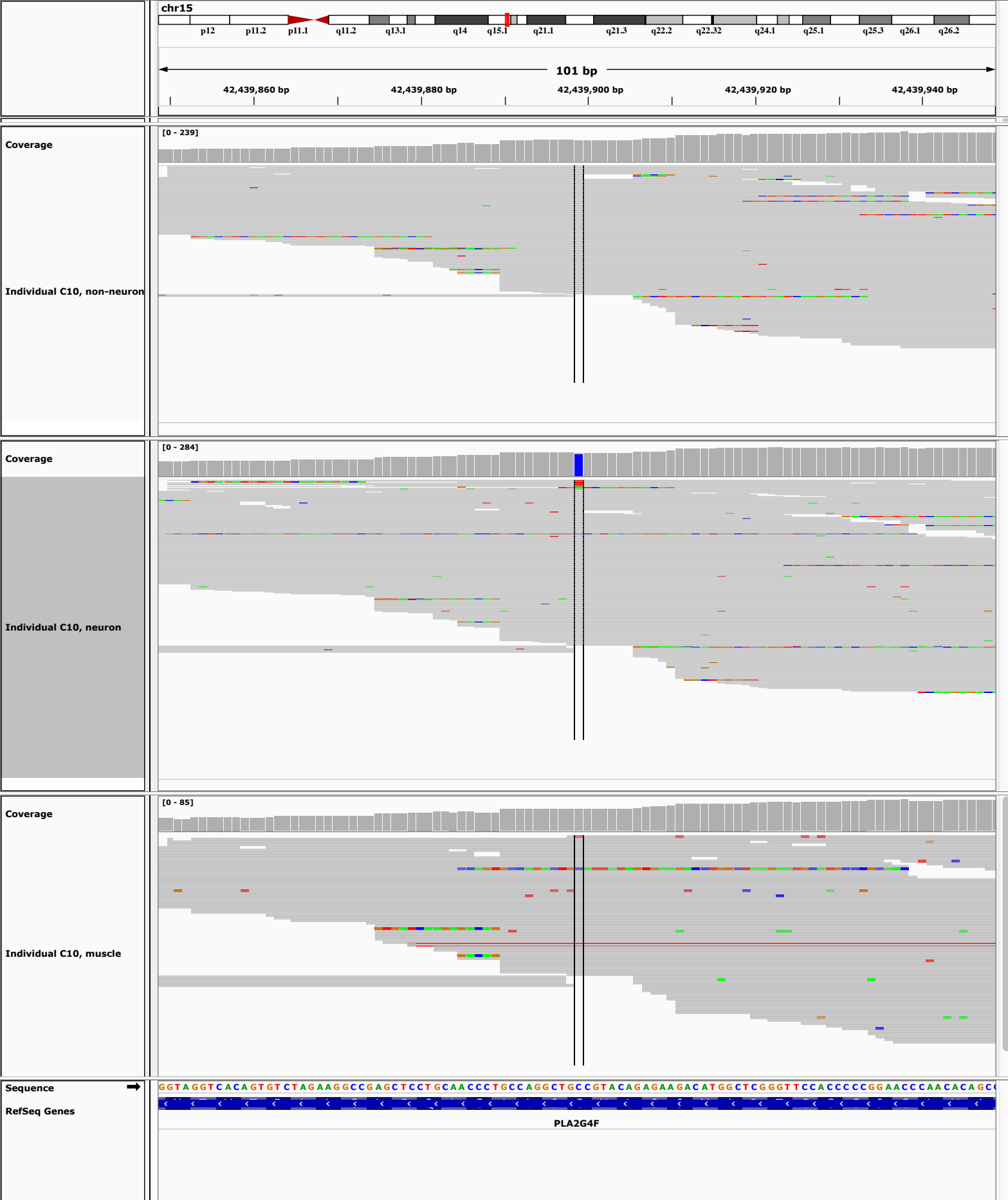

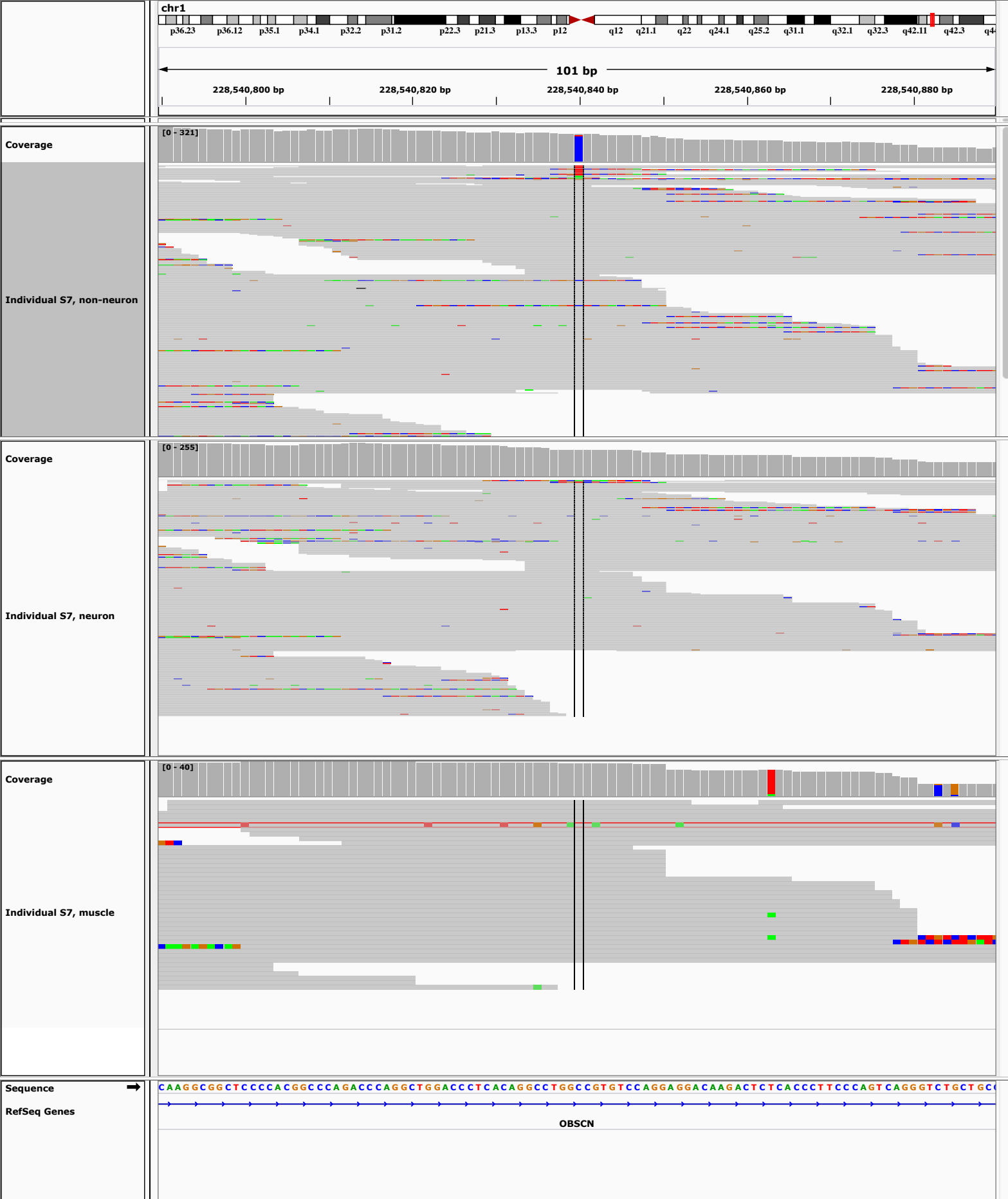



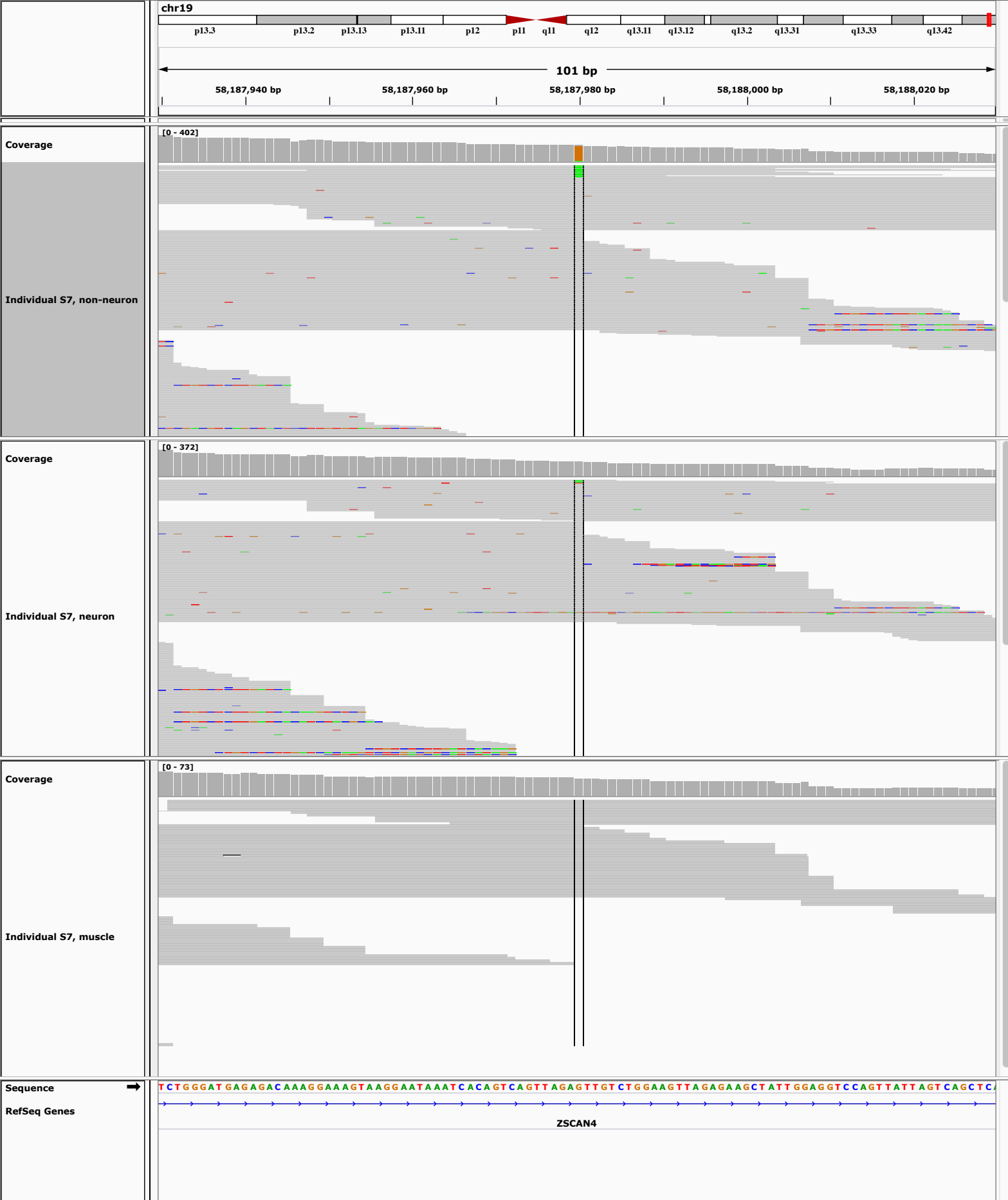

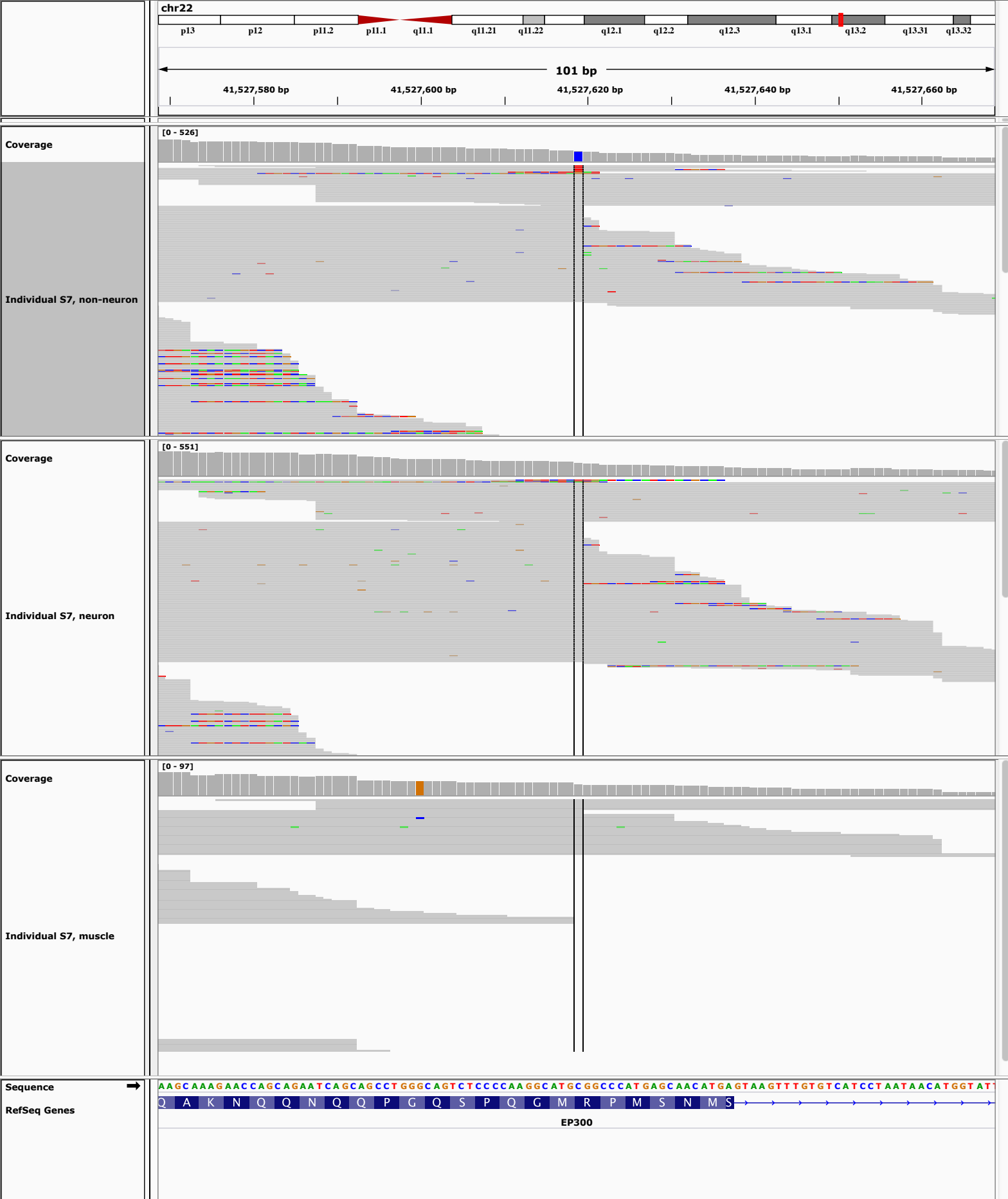

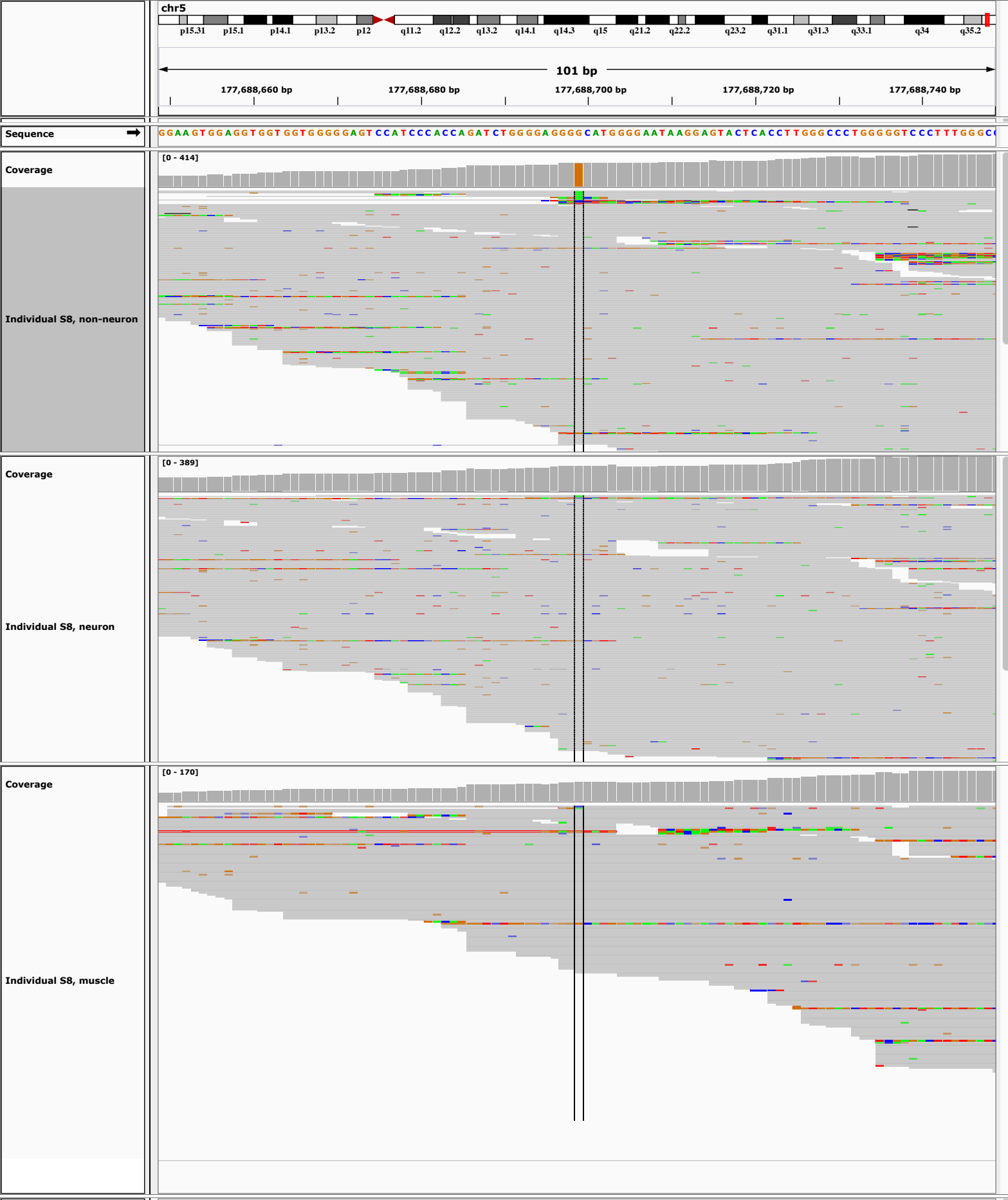

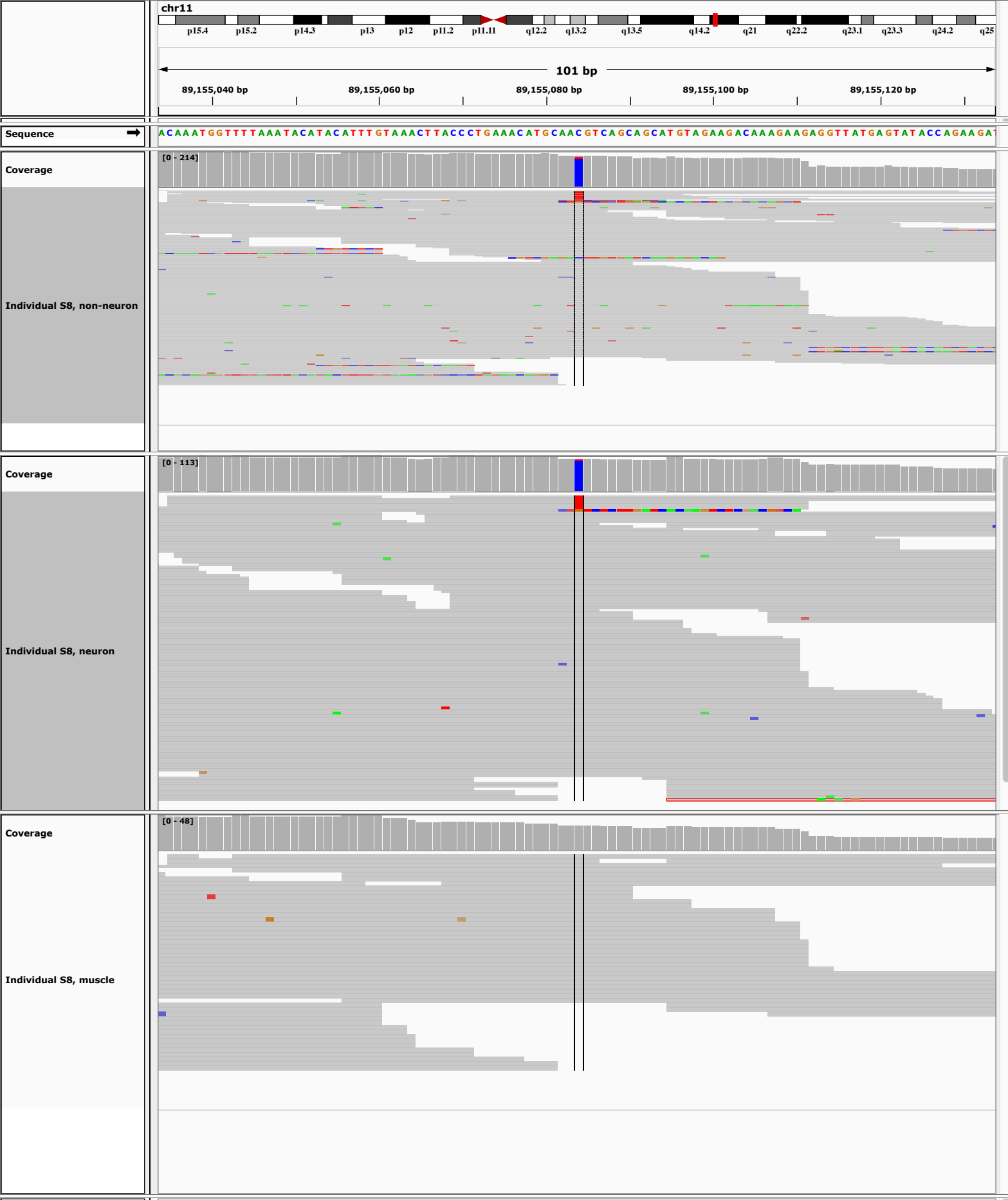

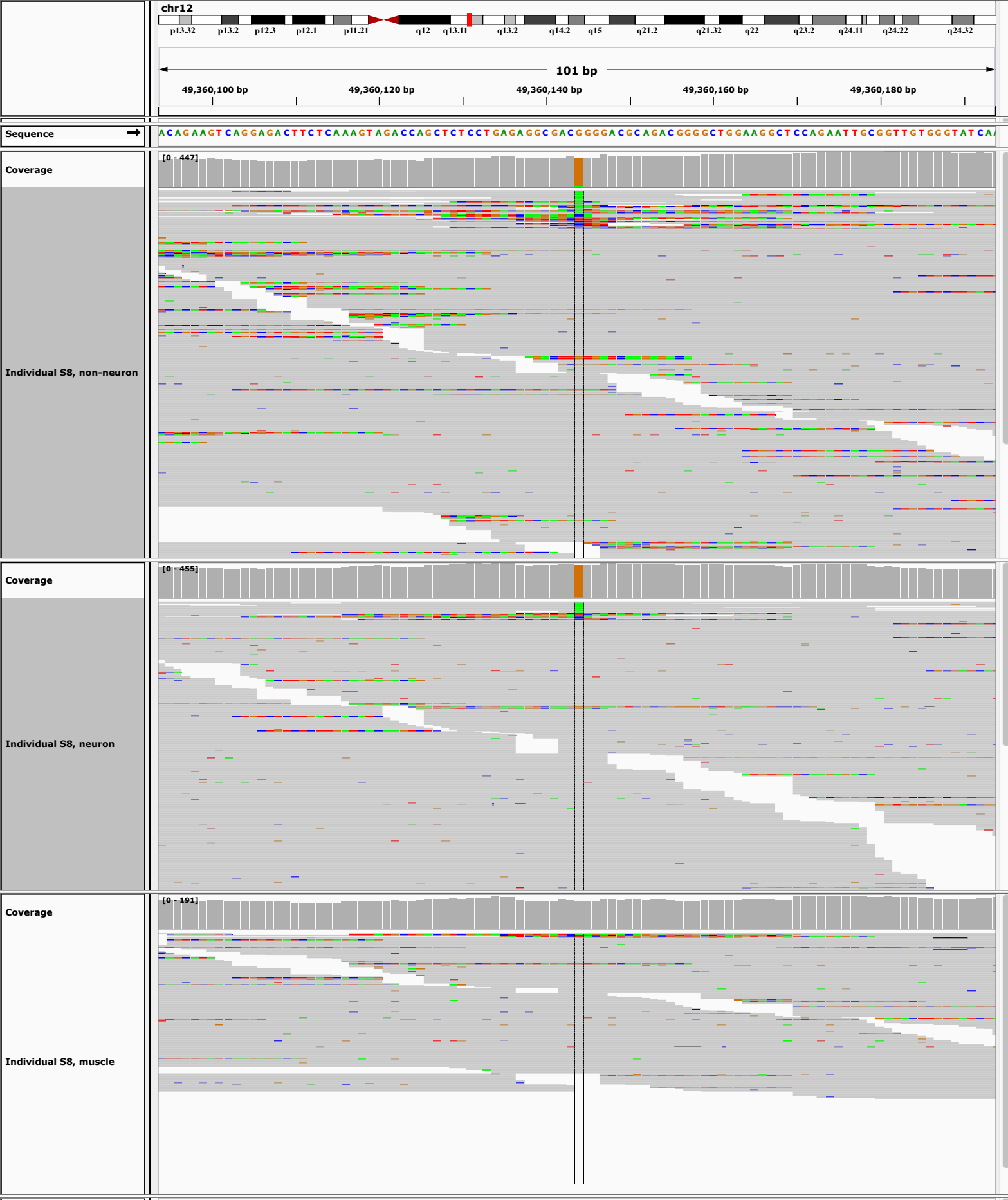

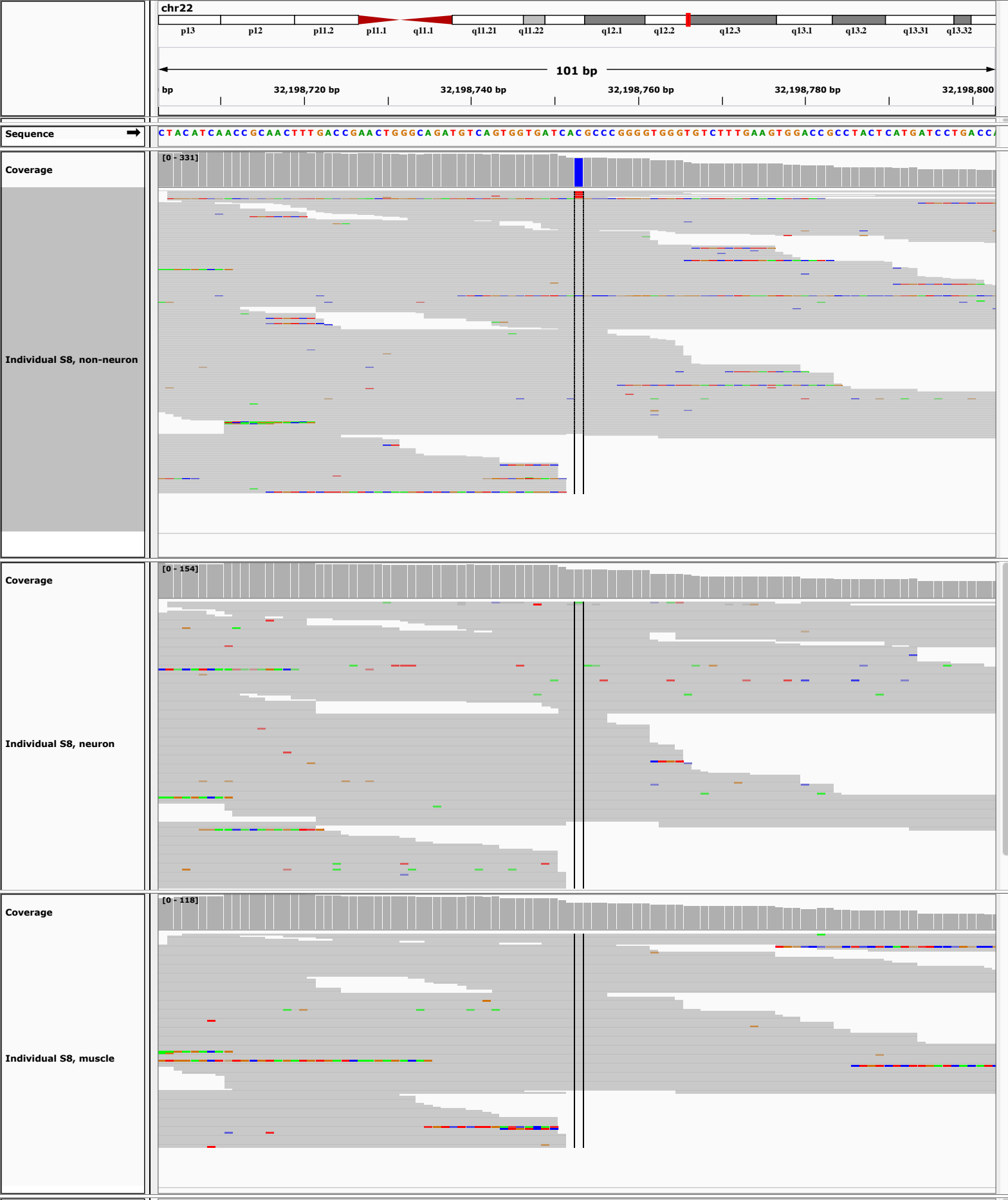

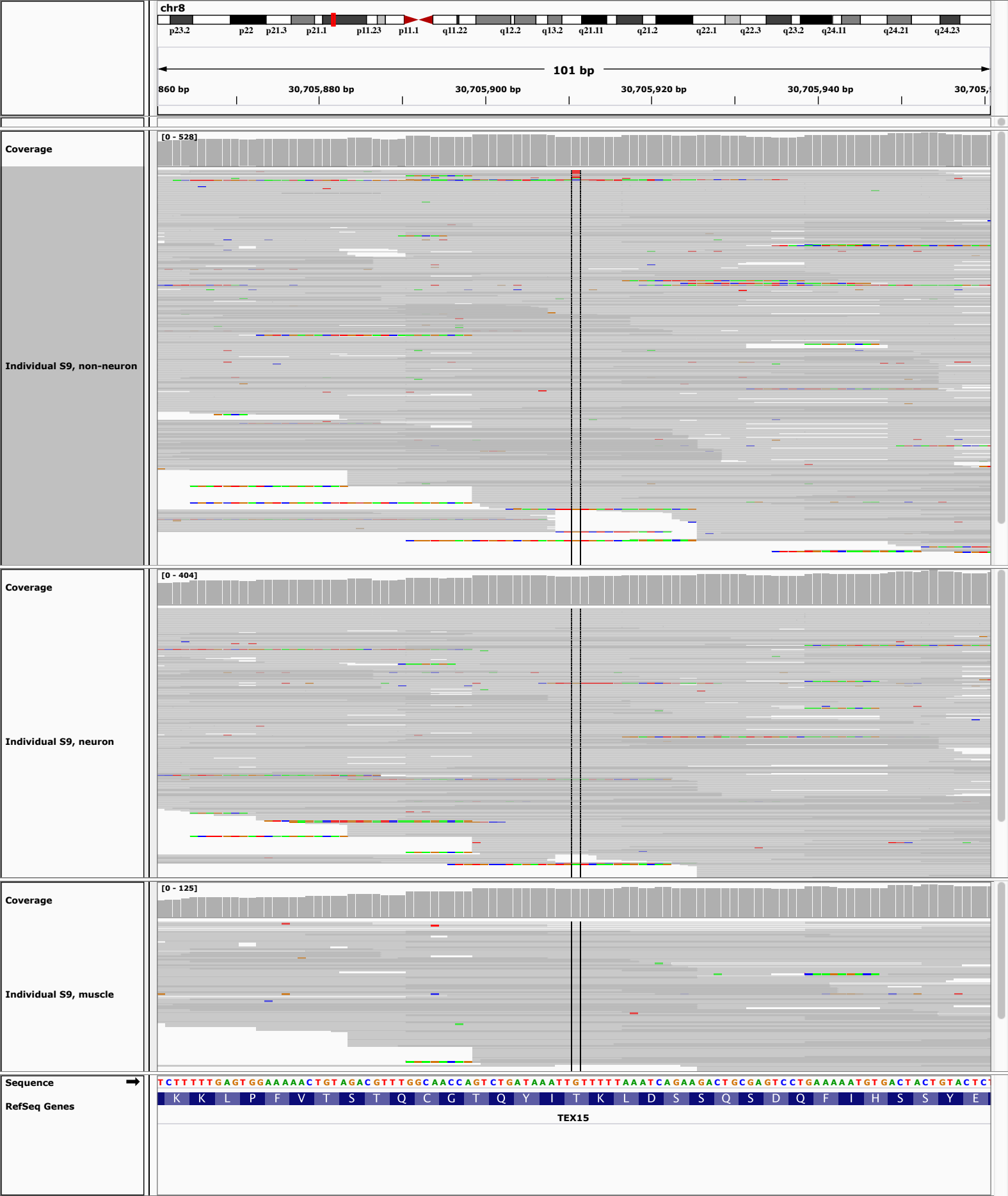

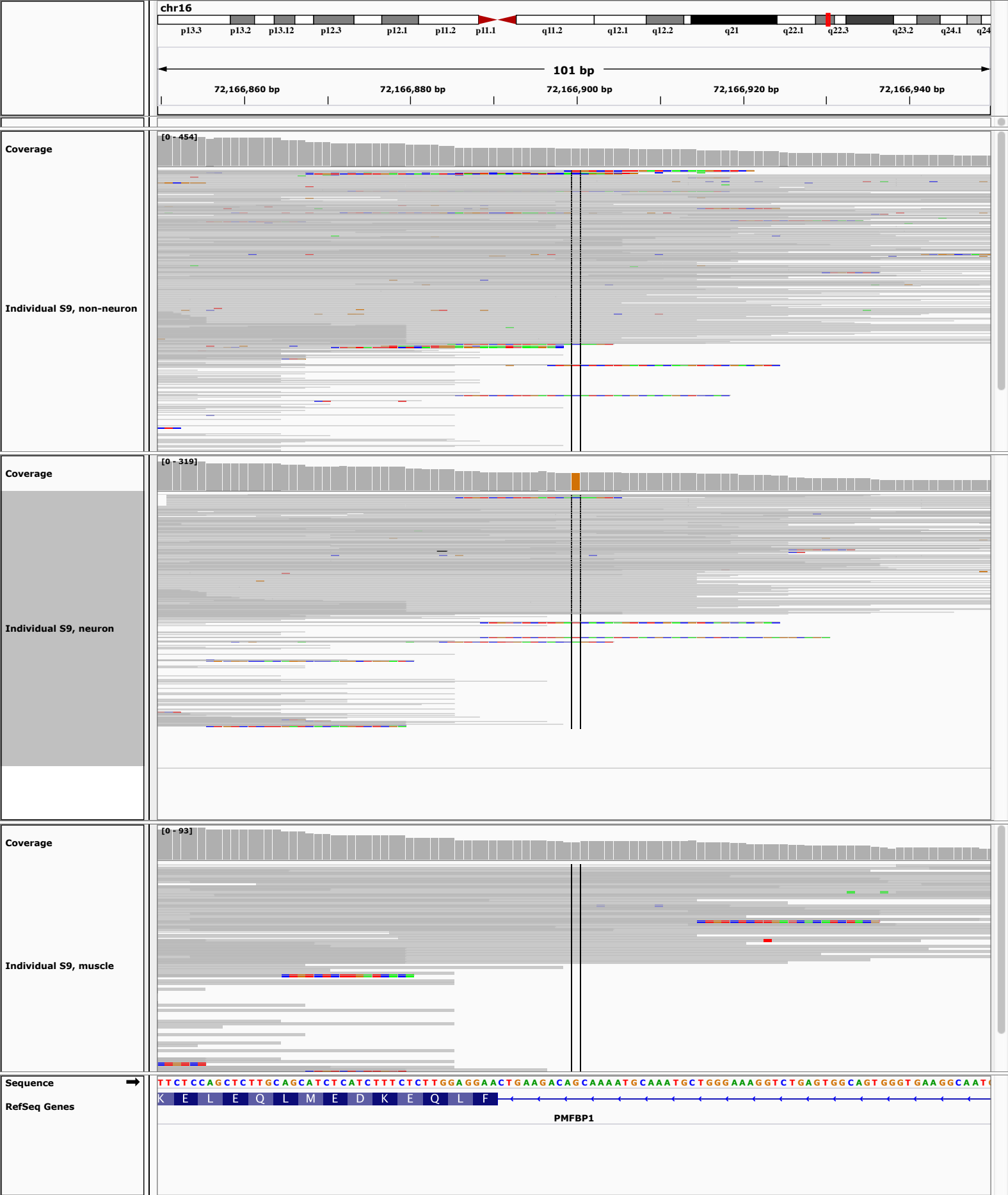

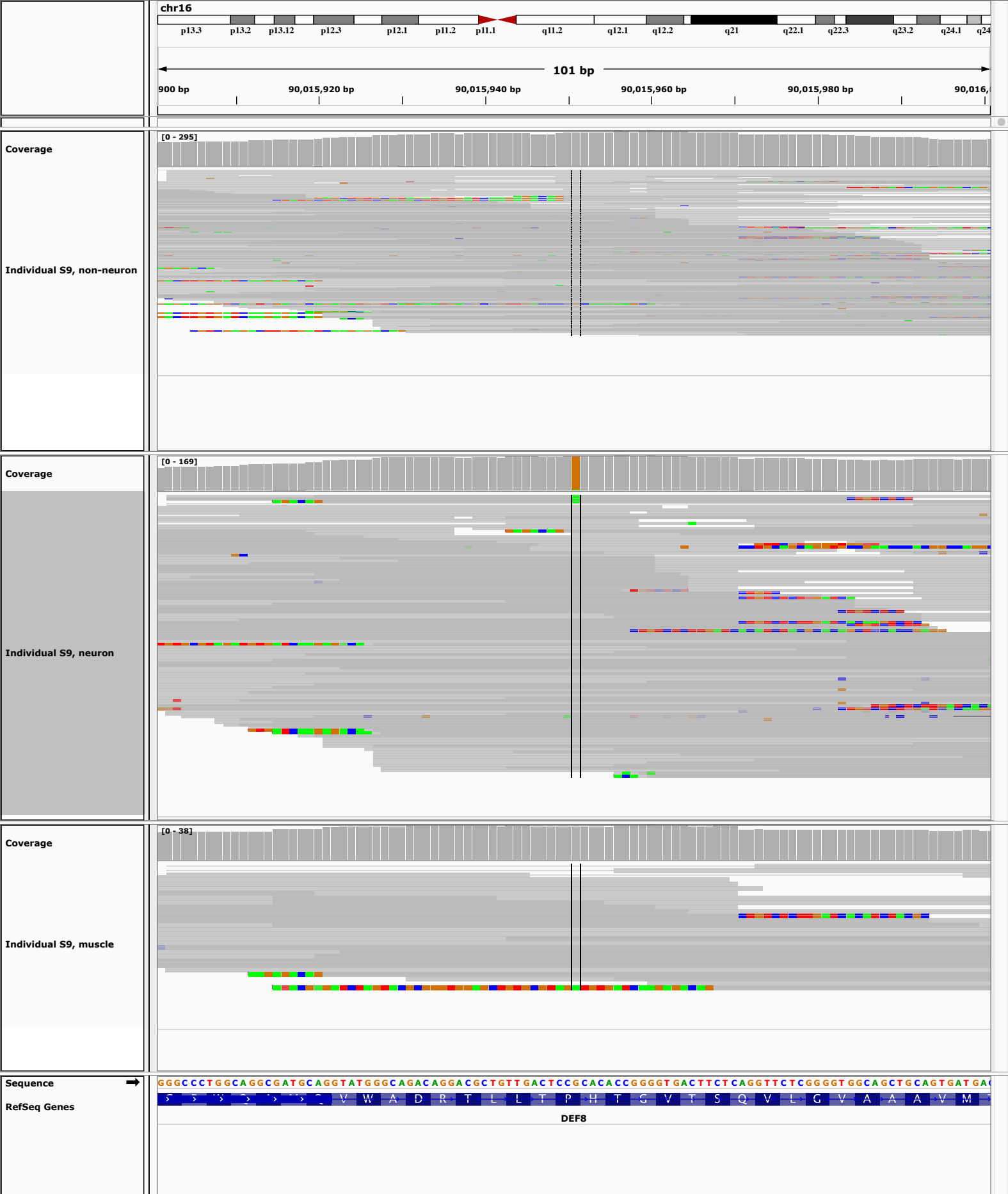

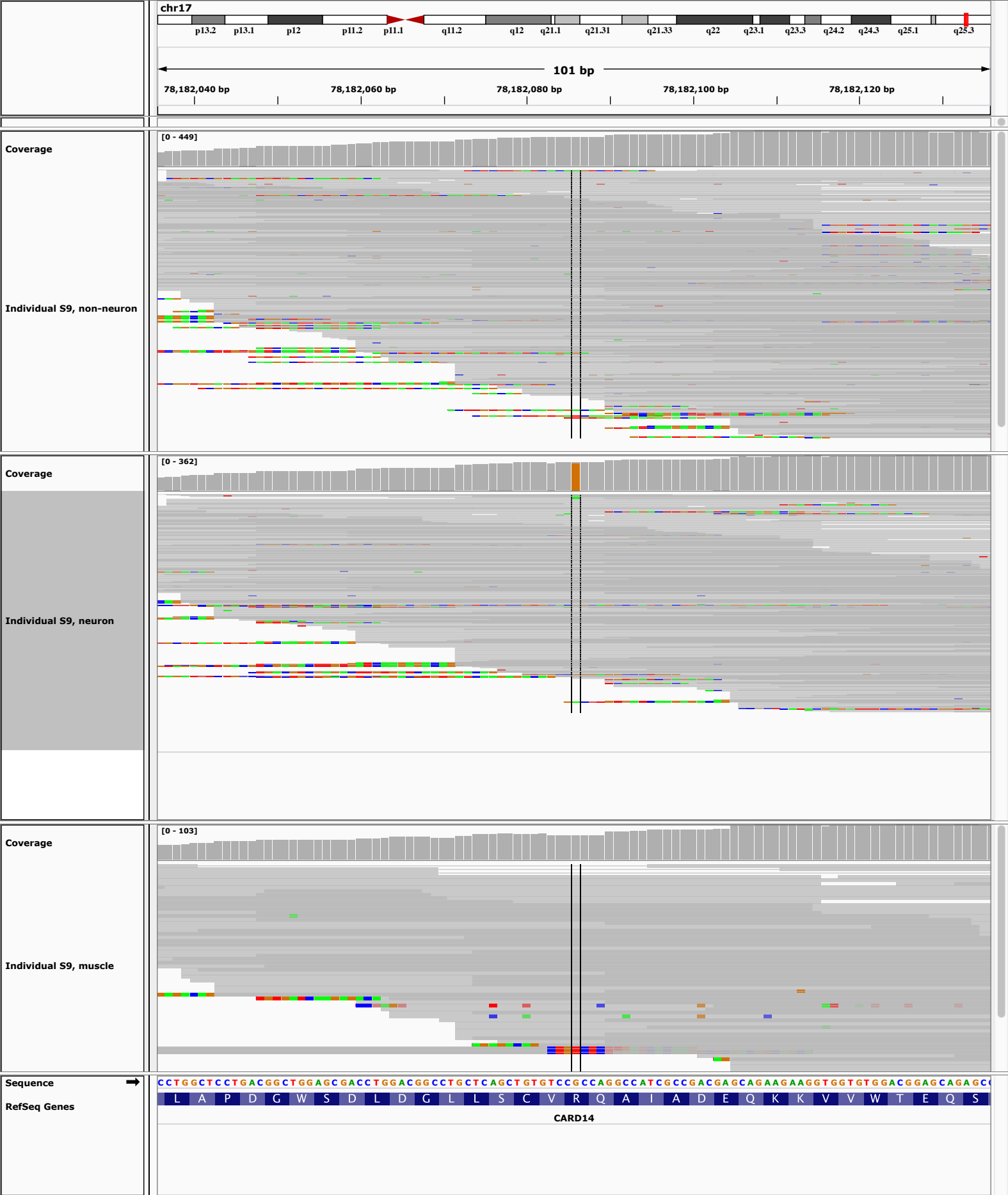

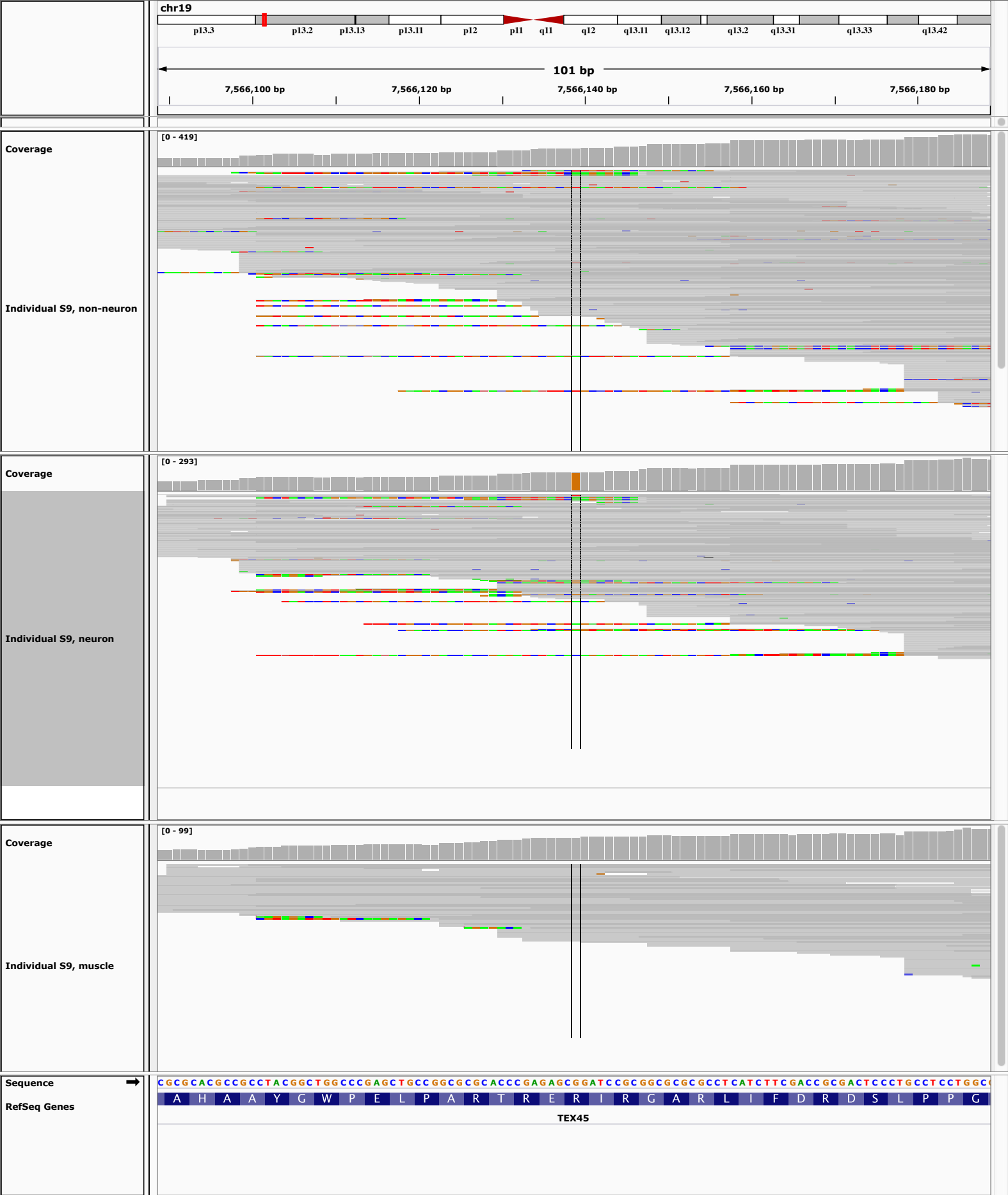

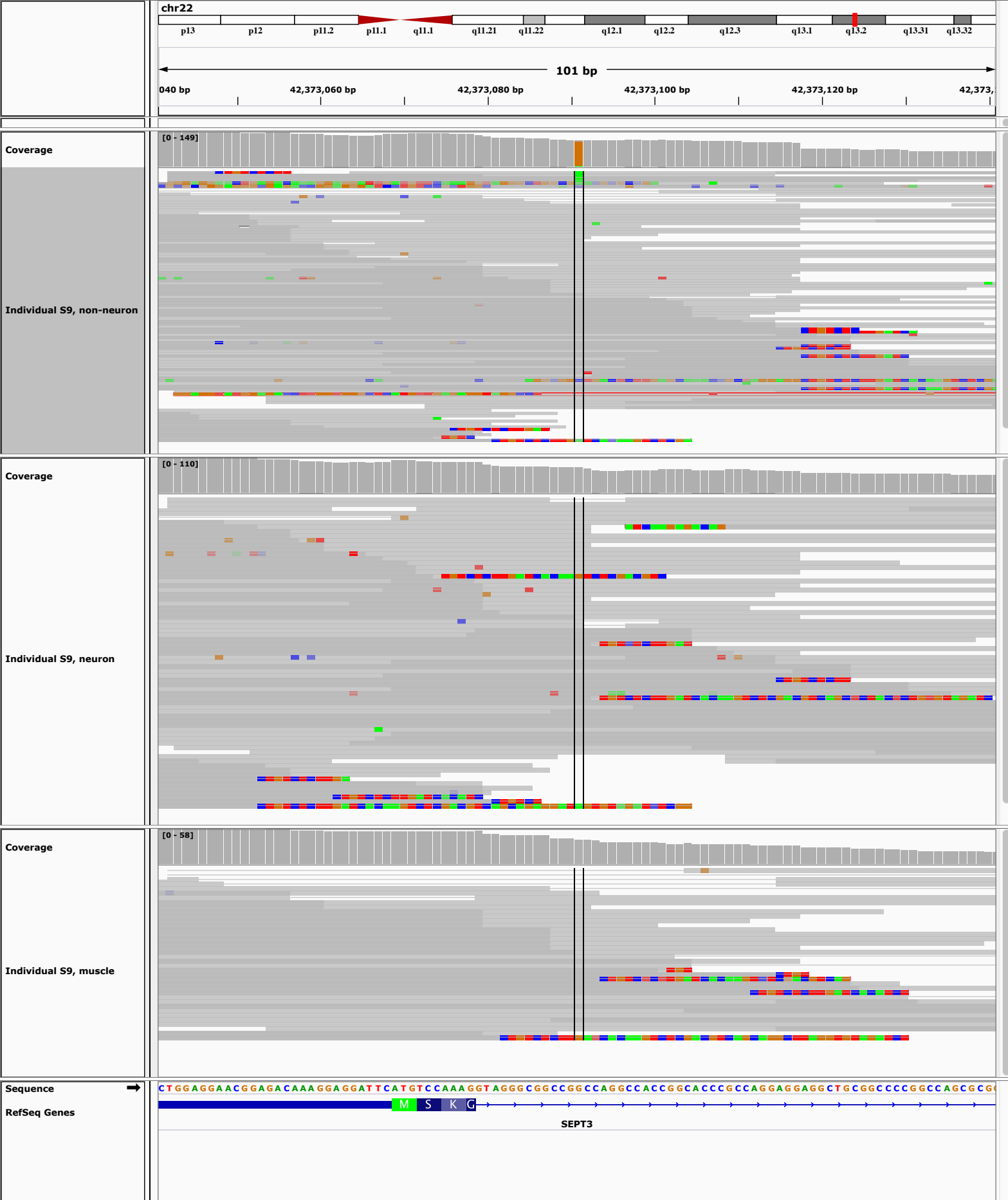

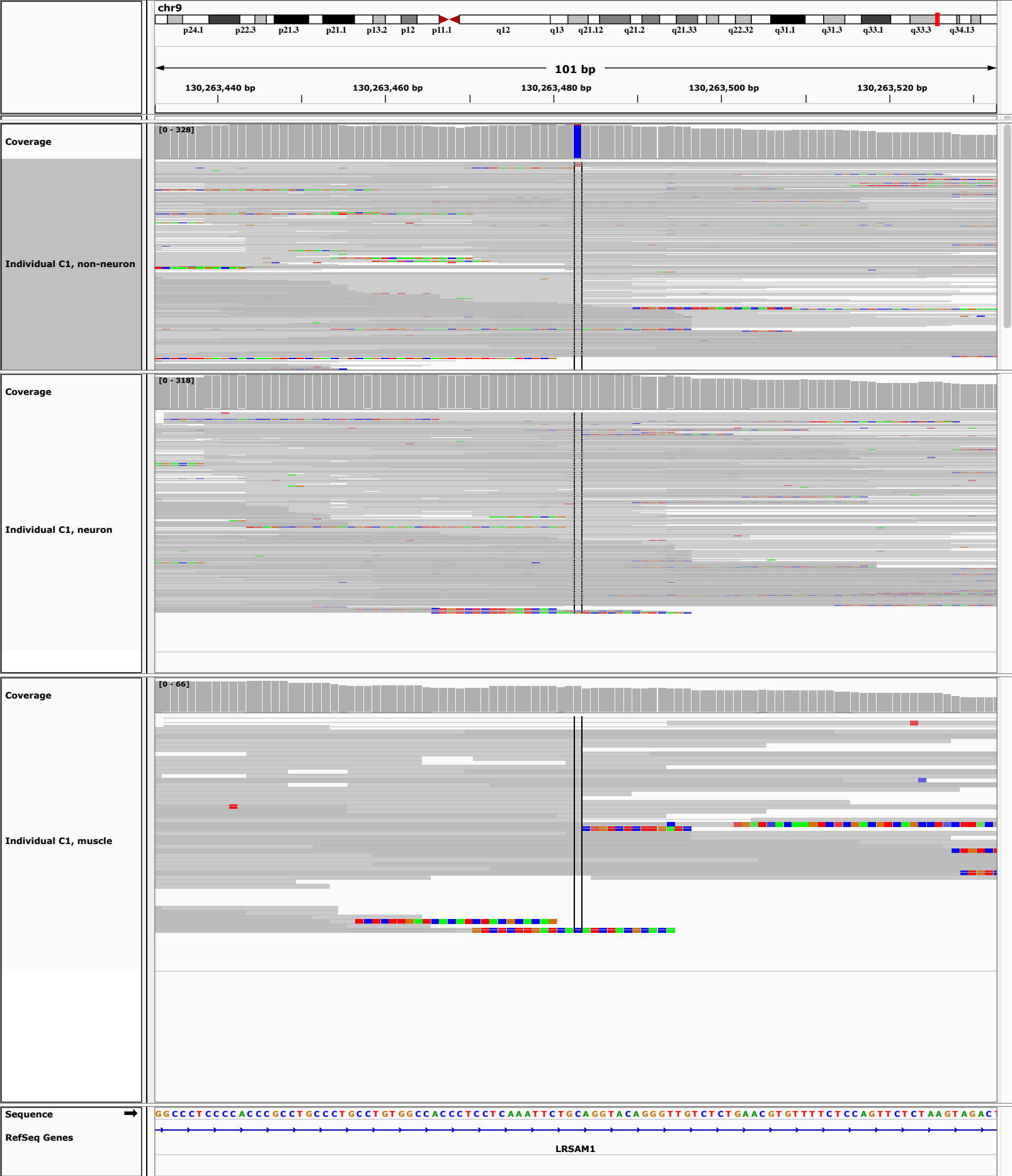

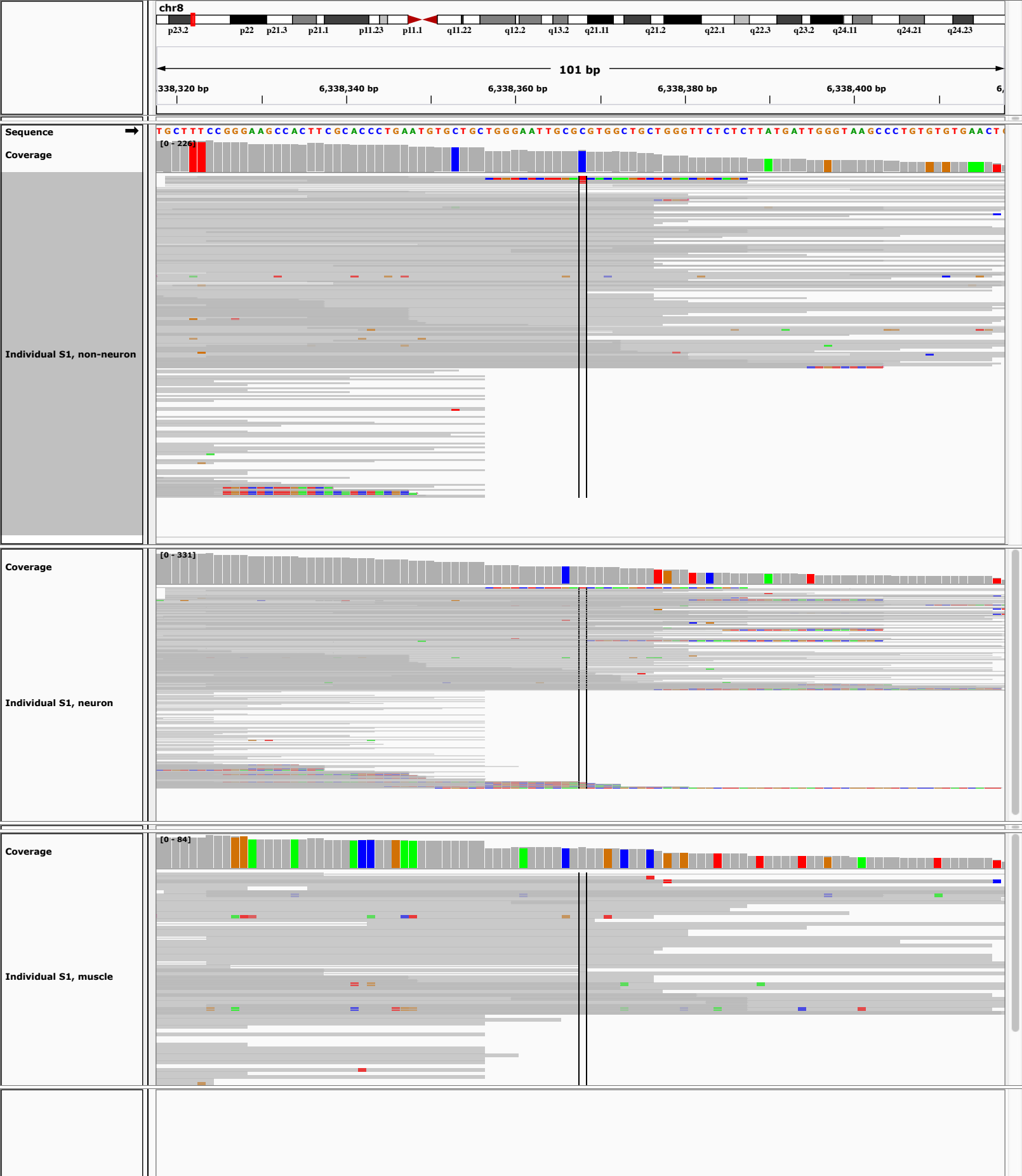

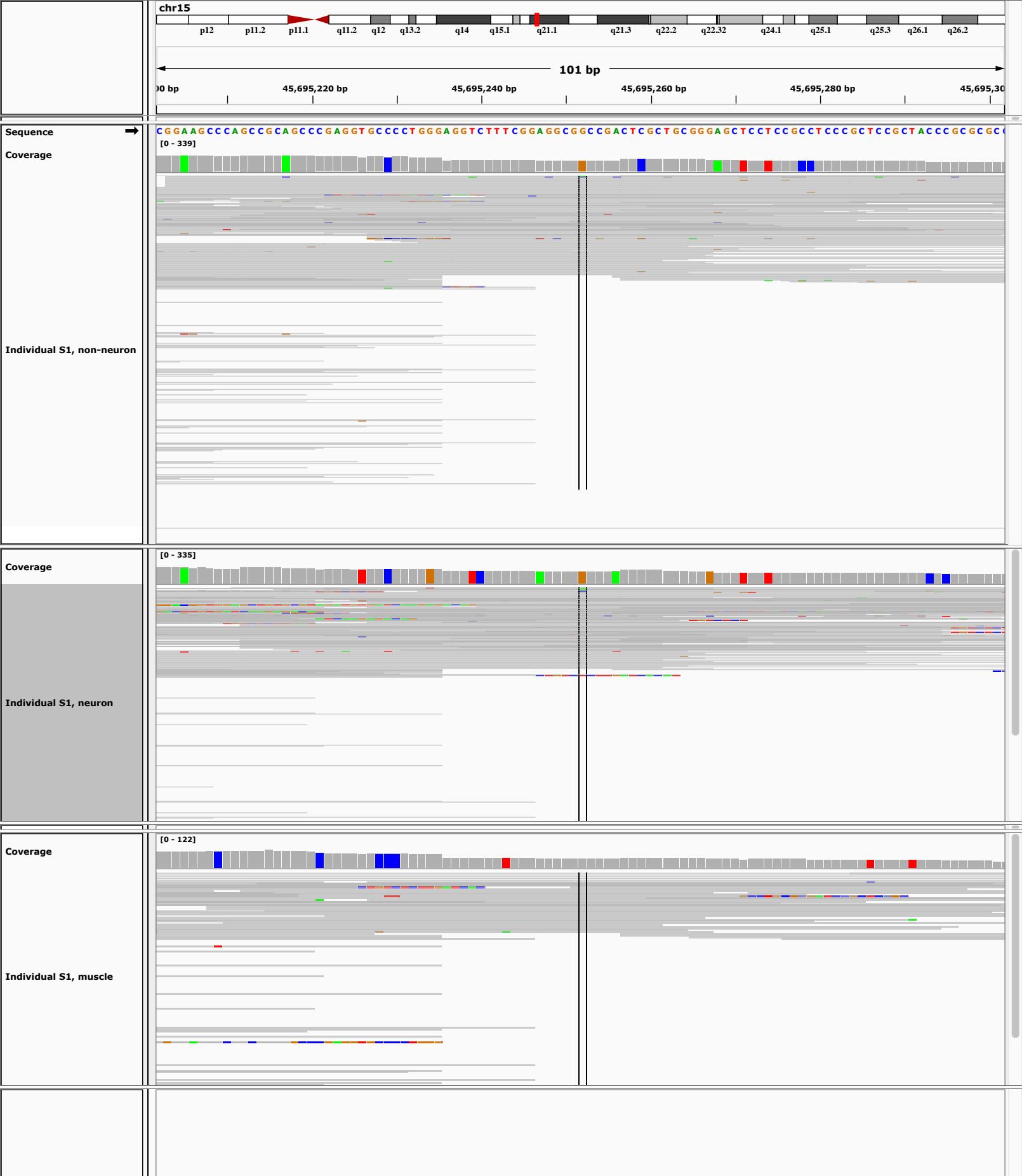

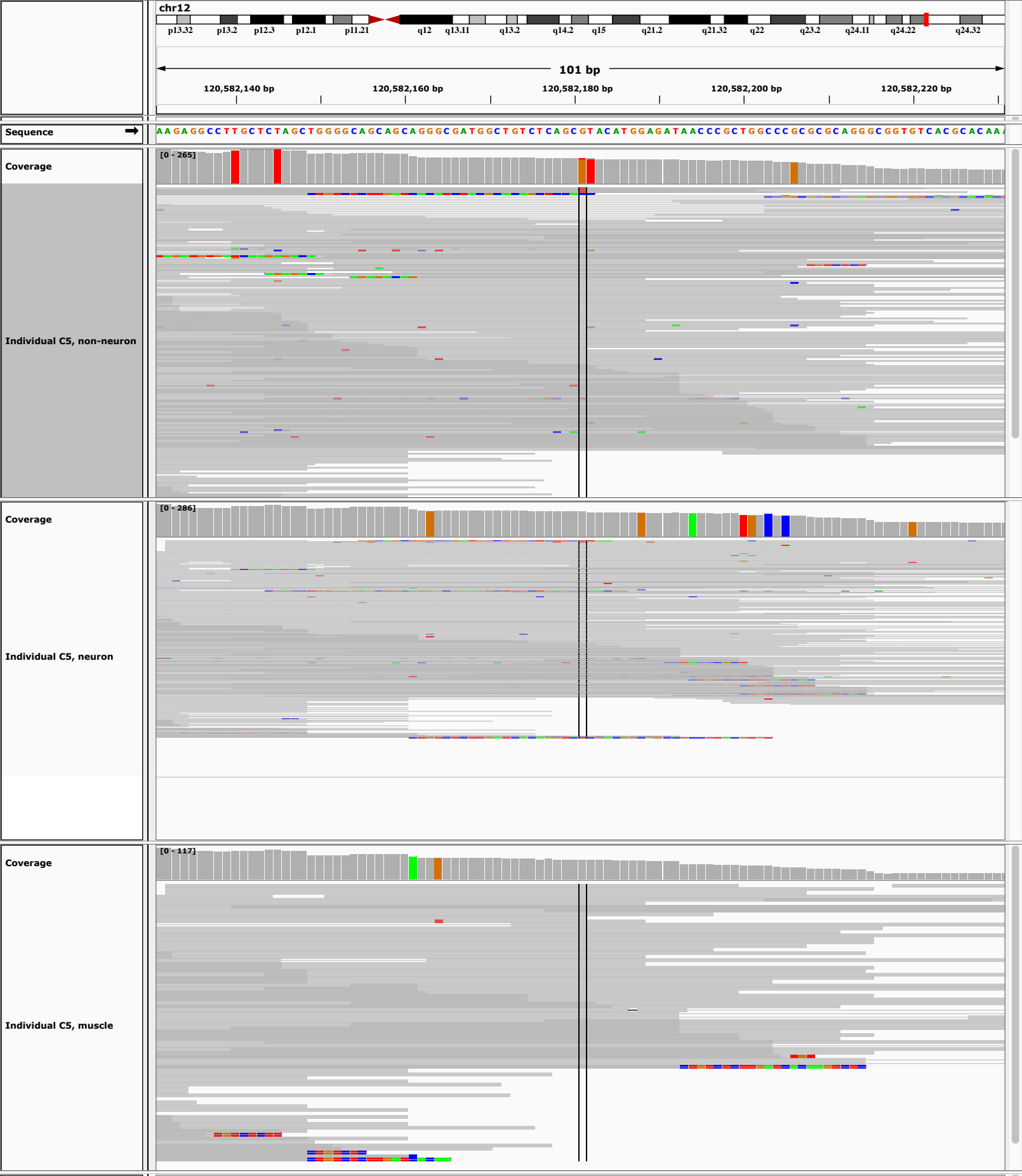

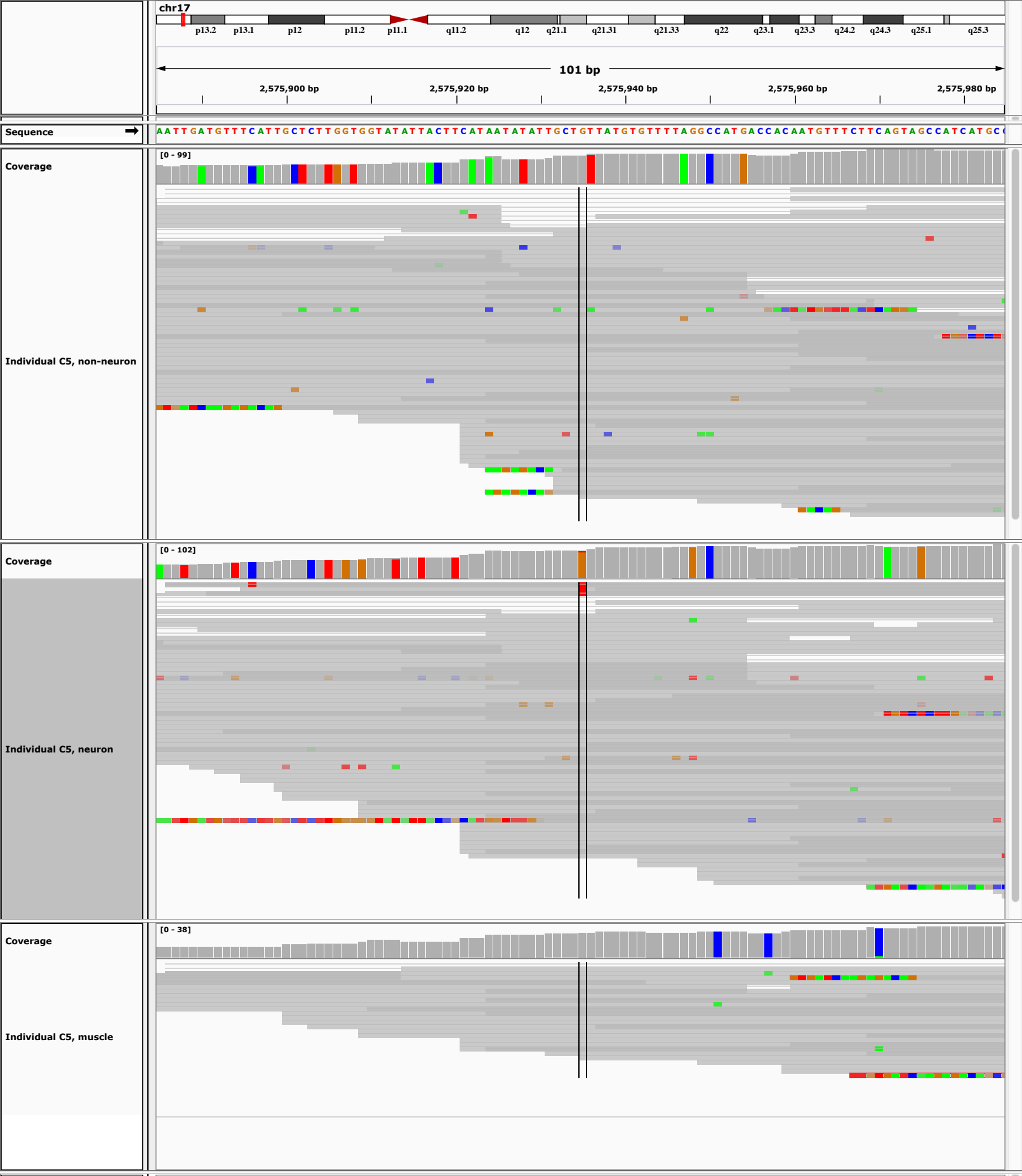

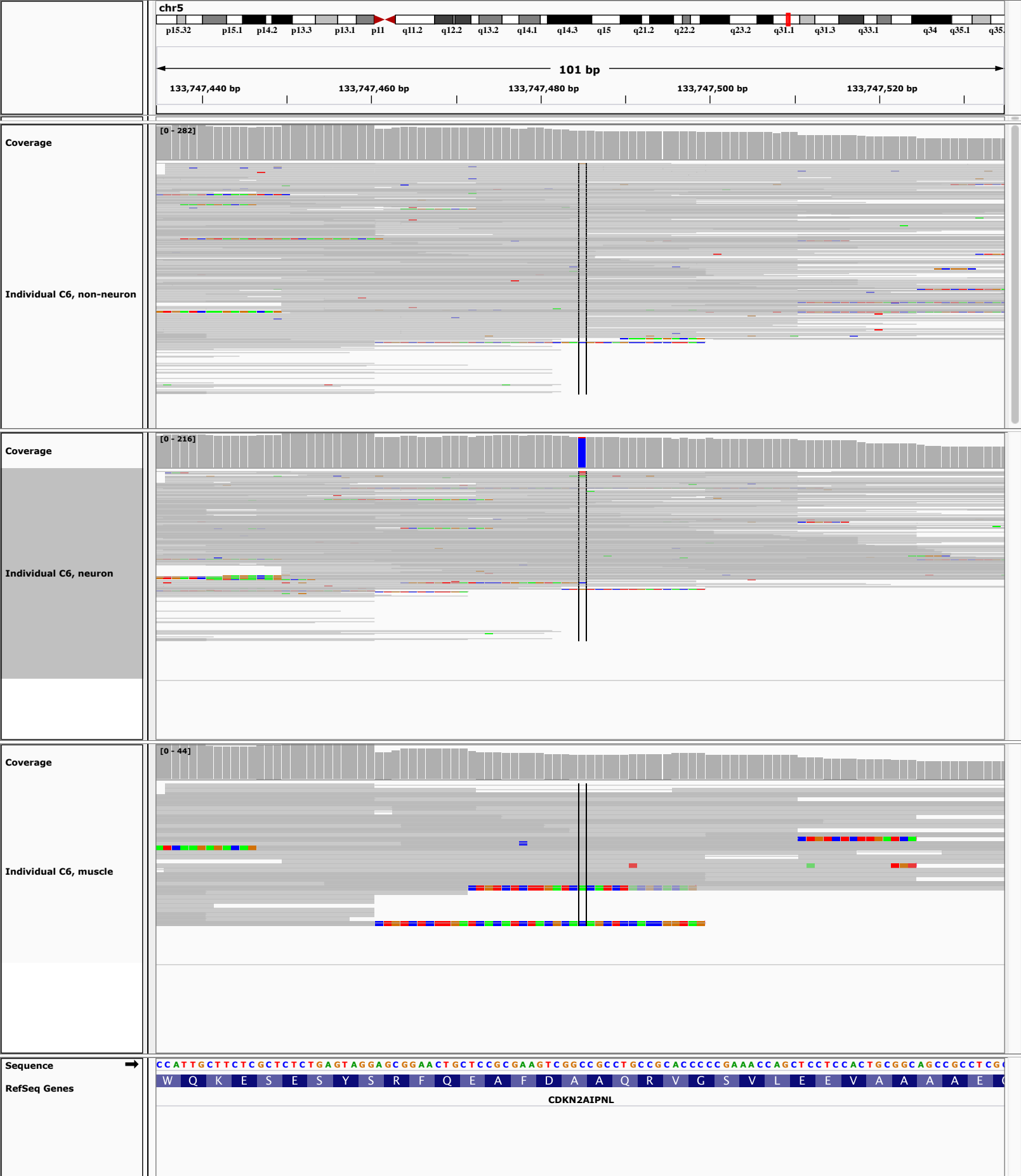

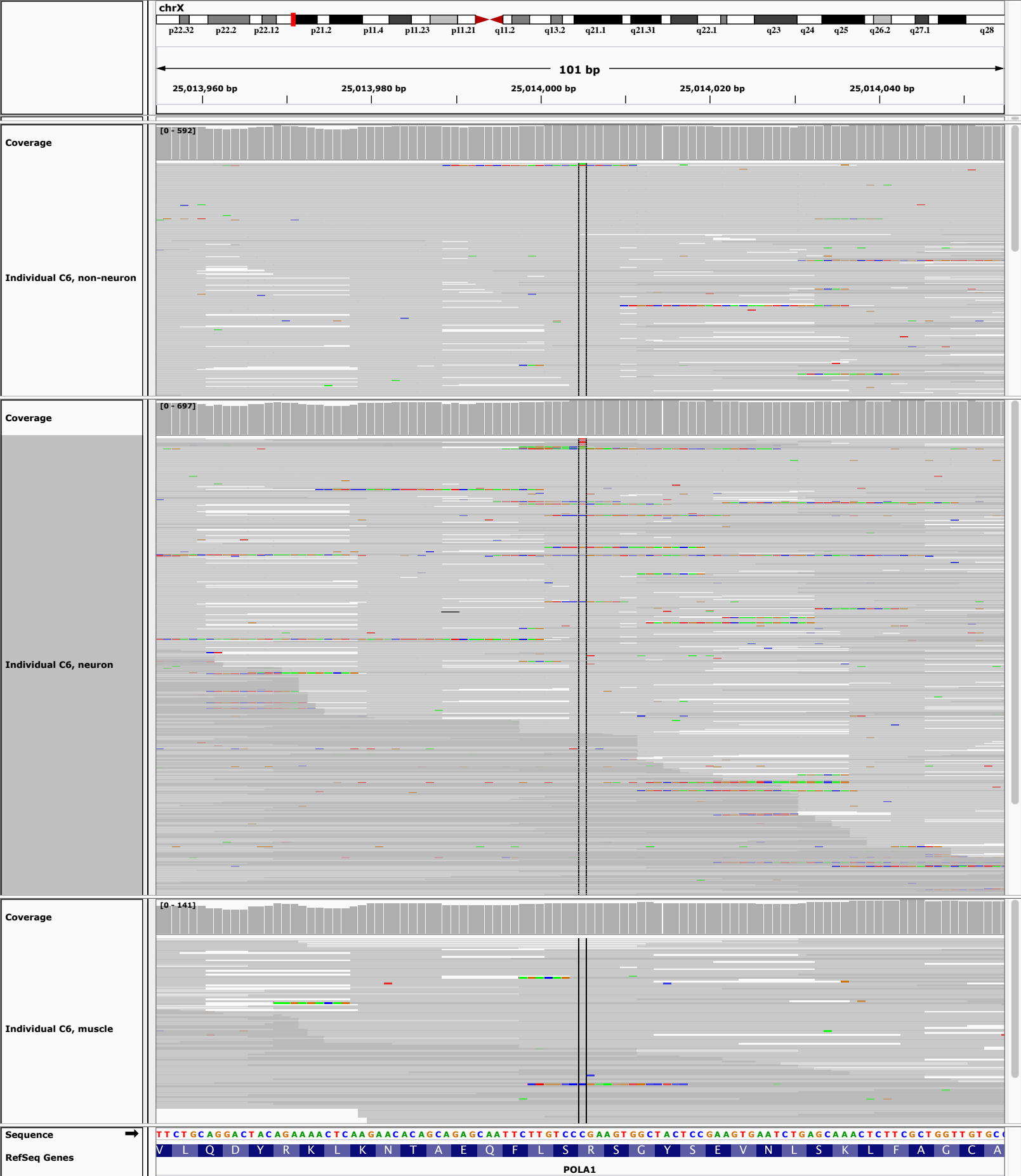

Supplement: Supplementary file 2 — Supplementary Figure 4 [file 41398_2018_342_MOESM2_ESM.pdf]
